# Characterisation of lamina I anterolateral system neurons that express Cre in a *Phox2a-Cre* mouse line

**DOI:** 10.1038/s41598-021-97105-w

**Published:** 2021-09-09

**Authors:** Wafa A. A. Alsulaiman, Raphaelle Quillet, Andrew M. Bell, Allen C. Dickie, Erika Polgár, Kieran A. Boyle, Masahiko Watanabe, R. Brian Roome, Artur Kania, Andrew J. Todd, Maria Gutierrez-Mecinas

**Affiliations:** 1 Institute of Neuroscience and Psychology, College of Medical, Veterinary and Life Sciences, University of Glasgow, Glasgow, G12 8QQ UK; 2 Department of Anatomy, Hokkaido University School of Medicine, Sapporo, 060-8638 Japan; 3 Institut de Recherches Cliniques de Montréal (IRCM), Montreal, QC H2W 1R7 Canada; 4 Spinal Cord Group, University of Glasgow, Sir James Black Building, Glasgow, G12 8QQ UK

**Keywords:** Sensory processing, Somatosensory system

## Abstract

A recently developed *Phox2a::Cre* mouse line has been shown to capture anterolateral system (ALS) projection neurons. Here, we used this line to test whether Phox2a-positive cells represent a distinct subpopulation among lamina I ALS neurons. We show that virtually all lamina I Phox2a cells can be retrogradely labelled from injections targeted on the lateral parabrachial area (LPb), and that most of those in the cervical cord also belong to the spinothalamic tract. Phox2a cells accounted for ~ 50–60% of the lamina I cells retrogradely labelled from LPb or thalamus. Phox2a was preferentially associated with smaller ALS neurons, and with those showing relatively weak neurokinin 1 receptor expression. The Phox2a cells were also less likely to project to the ipsilateral LPb. Although most Phox2a cells phosphorylated extracellular signal-regulated kinases following noxious heat stimulation, ~ 20% did not, and these were significantly smaller than the activated cells. This suggests that those ALS neurons that respond selectively to skin cooling, which have small cell bodies, may be included among the Phox2a population. Previous studies have defined neurochemical populations among the ALS cells, based on expression of Tac1 or Gpr83. However, we found that the proportions of Phox2a cells that expressed these genes were similar to the proportions reported for all lamina I ALS neurons, suggesting that Phox2a is not differentially expressed among cells belonging to these populations. Finally, we used a mouse line that resulted in membrane labelling of the Phox2a cells and showed that they all possess dendritic spines, although at a relatively low density. However, the distribution of the postsynaptic protein Homer revealed that dendritic spines accounted for a minority of the excitatory synapses on these cells. Our results confirm that Phox2a-positive cells in lamina I are ALS neurons, but show that the *Phox2a::Cre* line preferentially captures specific types of ALS cells.

## Introduction

The dorsal horn of the spinal cord receives sensory information from peripheral nerves, modulates this by means of complex local circuits, and transmits it to the brain through projection neurons^1,2^. Many dorsal horn projection neurons belong to the anterolateral system (ALS), so called because in the human spinal cord their axons cross the midline and ascend in the anterior part of the lateral funiculus. The importance of the ALS in pain perception is demonstrated by the loss of pain, as well as temperature and itch sensation, on the contralateral side after anterolateral cordotomy at the cervical level in patients^3,4^. The constituent neurons of the ALS project to a variety of supraspinal targets, including several thalamic nuclei, the periaqueductal grey matter (PAG), the lateral parabrachial area (LPb) and various nuclei in the medulla^2,5–8^.

Retrograde tracing studies have revealed a relatively high concentration of ALS neurons in lamina I and the lateral spinal nucleus (LSN), as well as cells located throughout the deeper dorsal horn (laminae III–VI), the area around the central canal and the ventral horn^8–12^. The ALS neurons in lamina I have attracted particular attention because of their proposed role in pathological pain states. This was initially based on the findings that many of these cells expressed high levels of the neurokinin 1 receptor (NK1r)^2,13,14^, and that ablation of NK1r-expressing cells by intrathecal administration of substance P conjugated to saporin suppressed the exaggerated responses seen in models of both neuropathic and inflammatory pain^15^. It was subsequently shown that changes in the response properties of lamina I ALS neurons recorded in vivo matched the increased reflex responses seen in neuropathic pain models^16,17^. Both electrophysiological mapping of the axons of lamina I projection cells^18^ and retrograde tracing studies^19^ have shown that individual lamina I neurons in the rat spinal cord send collateral branches to more than one brainstem target. In addition, we have demonstrated that virtually all lamina I ALS cells in the rat can be retrogradely labelled from a single tracer injection into the LPb^20,21^.

Electrophysiological and imaging studies in various species have shown that many lamina I ALS neurons can be activated by more than one type of cutaneous stimulus, and that they are diverse in terms of response properties^16,17,22–27^. Most of these cells respond to noxious mechanical and thermal stimuli, with some additionally responding to innocuous mechanical stimuli or to itch-inducing chemicals, and some being selectively activated by innocuous cooling. The heterogeneity of lamina I ALS neurons has led to several recent attempts to define populations among these cells, by identifying molecular-genetic markers that can be used to target specific classes^28–30^. Notably, Choi et al.^28^ have identified two largely non-overlapping groups based on mouse lines in which Cre recombinase fused to the ligand-binding domain of the estrogen receptor (CreERT2) was knocked into either Tacr1 (the gene for the NK1 receptor) or Gpr83 (which codes for another G-protein-coupled receptor).

Roome et al.^7^ have recently developed a BAC transgenic mouse line in which Cre is expressed under control of the promoter for the transcription factor Phox2a (*Phox2a::Cre*). After crossing this with a Cre-dependent tdTomato reporter line, they showed that tdTomato labelling was virtually restricted to ALS neurons in the spinal cord, and captured around half of those in lamina I. In the present study, we have crossed the Phox2a::Cre mouse with Cre-dependent reporter lines to test whether this strategy preferentially targets specific types of lamina I ALS neuron, defined by neurochemistry or response to noxious stimuli. In addition, we have used this approach to investigate the distribution of excitatory synapses on these cells.

## Results

### Distribution of Phox2a cells in lamina I and their relation to spinoparabrachial neurons

We crossed *Phox2a::Cre* and *Rosa26*^*LSL-tdTomato*^ lines to generate *Phox2a::Cre;Rosa26*^*LSL-tdTomato*^ mice, in which most cells that express Phox2a at any stage during development are permanently labelled with tdTomato. The pattern of tdTomato expression in the spinal cord was the same as described previously^7^, and examples from the lumbar region are shown in Fig. S1. TdTomato-positive cells were relatively numerous in lamina I, and were present at lower density in deeper dorsal horn laminae (III–VI), the LSN and the area around the central canal. A few cells were seen in the ventral horn. TdTomato-positive cells in laminae III and IV often had dorsally directed dendrites that extended as far as lamina I, corresponding to "antenna-type" projection neurons that have been identified in both rat and mouse^9,31,32^.

**Figure 1:**
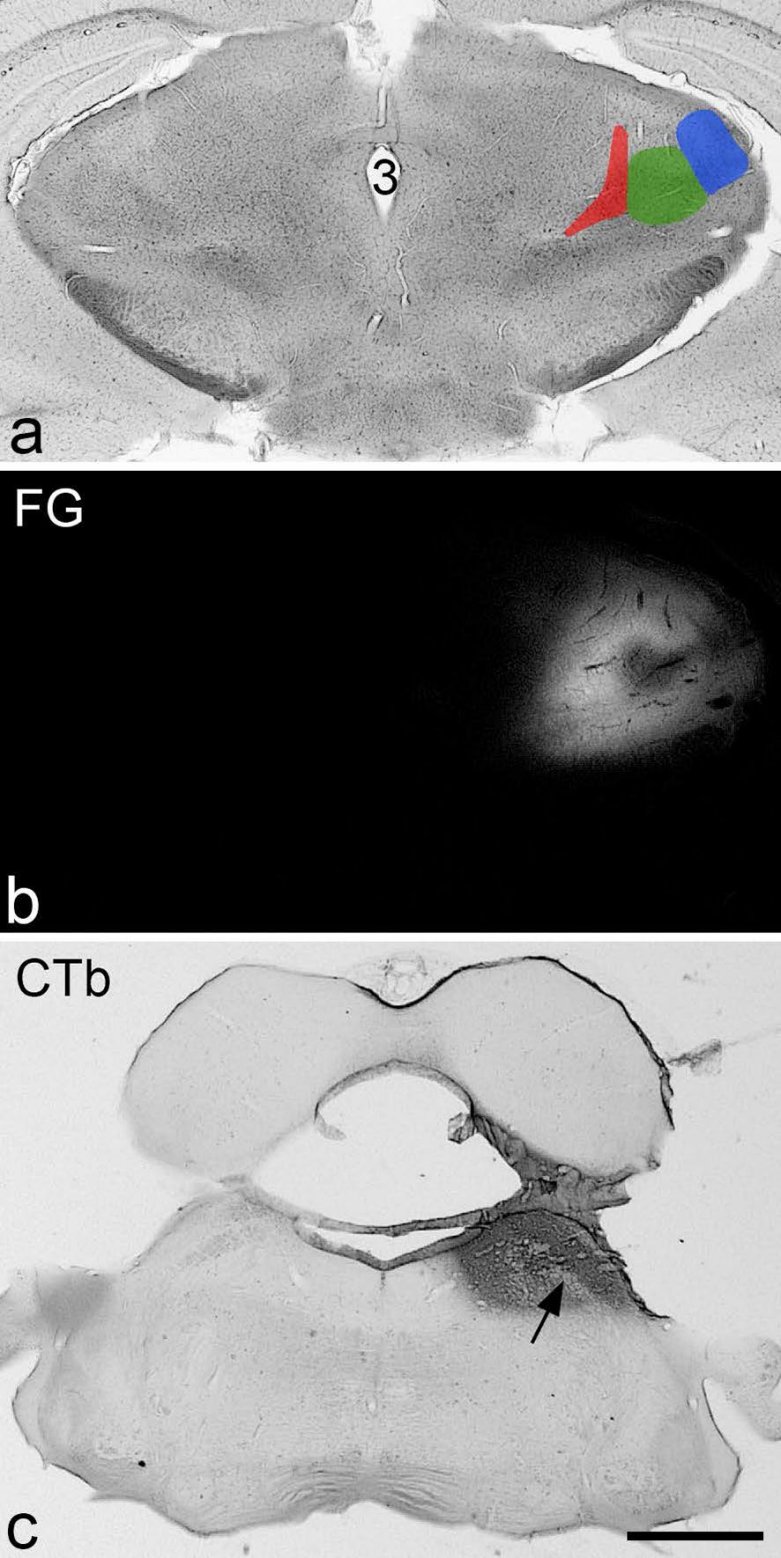
Representative injection sites in the thalamus and brainstem from one of the experiments (#6). (**a**,**b**) Brightfield and fluorescence and images of a section through the diencephalon reveal the extent of the Fluorogold (FG) injection. Areas shown in red, green and blue correspond to the posterior triangular nucleus of the thalamus, and the medial and lateral geniculate nuclei, respectively. The 3rd ventricle is also indicated (3). (**c**) A section through the pons that has been reacted by an immunoperoxidase method to reveal cholera toxin B subunit (CTb). The arrow indicates the position of the superior cerebellar peduncle. Scale bar (**a–c**) = 1 mm.

**Figure 2:**
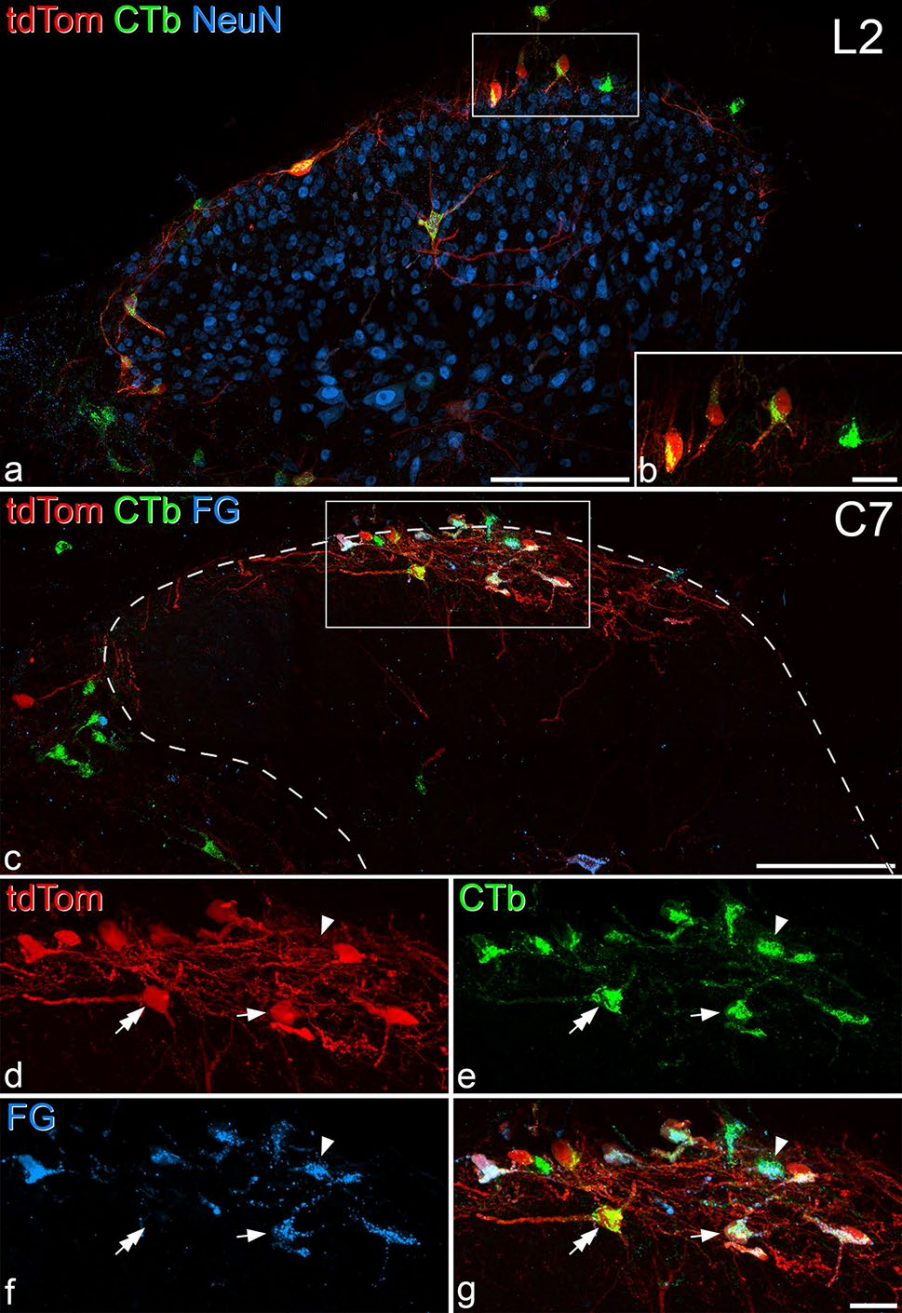
The relationship between retrograde labelled and Phox2a-positive cells. (**a**) Transverse section through the L2 segment of experiment #4, showing the side contralateral to the cholera toxin B subunit (CTb) injection into the lateral parabrachial area. The section has been immunostained to reveal tdTomato (tdTom, red), CTb (green) and NeuN (blue). Several retrogradely labelled neurons are visible, and most of these are in lamina I. Several of these are also tdTom-positive, and therefore appear yellow. The box shows the region corresponding to (**b**). (**b**) Part of lamina I from the same section showing 4 retrogradely-labelled lamina I cells, which contain CTb in the cytoplasm. The one on the right side is negative for tdTom, while the other three are tdTom-positive. (**c**) Transverse section through the C7 segment of experiment #6, showing the side contralateral to the CTb and Fluorogold (FG) injections. The section has been stained to reveal tdTom (red), CTb (green) and FG (blue). There is a cluster of retrogradely labelled cells, many of which are tdTom-positive in the central part of lamina I. The dashed line shows the outline of the grey matter, and the box indicates the region shown at higher magnification in (**d**–**g**). (**d**–**g**) Separating the individual colours reveals several patterns of co-localisation, including CTb-positive cells that also contain FG and tdTom (example shown with arrow). There are also tdTom-negative cells that are labelled with both CTb and FG (example shown with arrowhead) and CTb-labelled tdTom-positive cells that lack FG (example shown with double arrow). All images are from maximum intensity projections of confocal image scans (1 μm z-separation) through the full thickness of the 60 μm sections. In both (**a,c**), medial is to the right. Scale bars: (**a,c**) = 100 μm; (**b,d**–**f**) = 20 μm.

**Figure 3:**
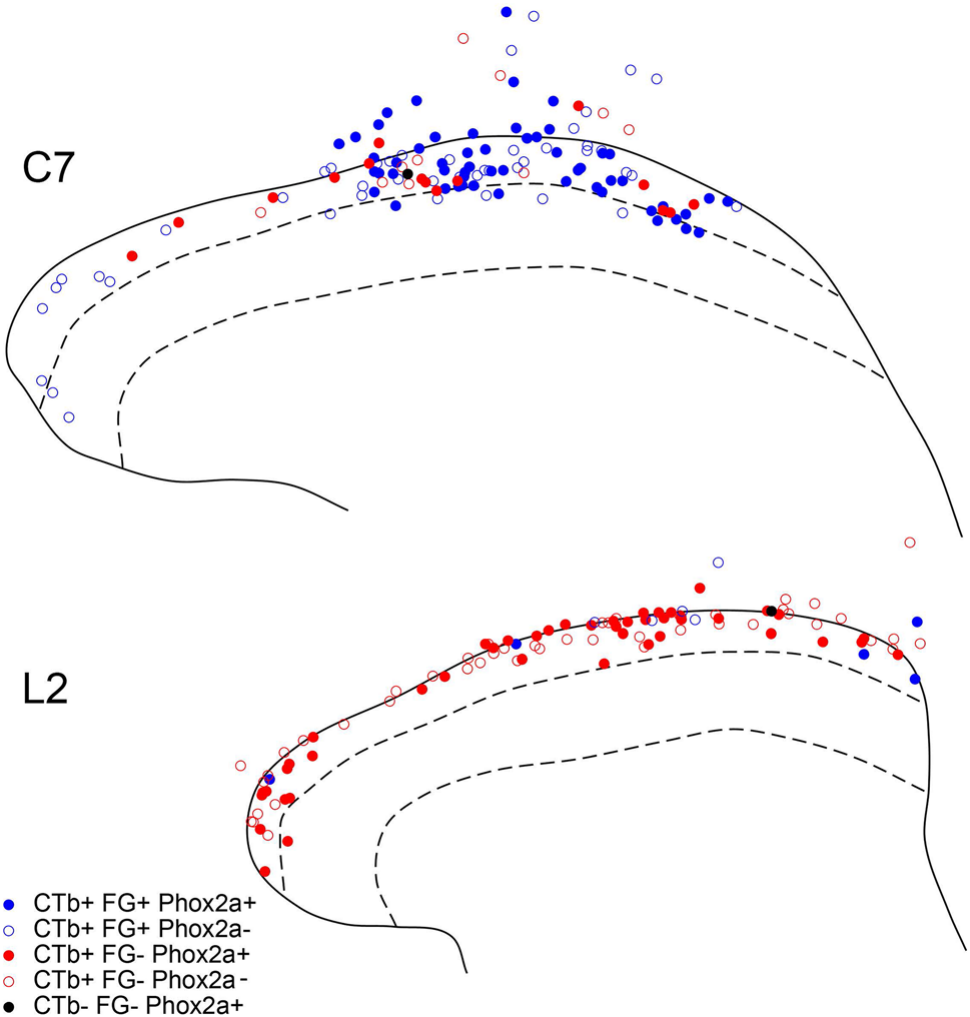
Plots showing the distribution of retrogradely-labelled and tdTomato-positive cells in the C7 and L2 segments from experiment #6, in which injections of cholera toxin B subunit (CTb) and Fluorogold (FG) were targeted on the lateral parabrachial area and thalamus, respectively. tdTomato-positive and -negative cells are shown with solid and open symbols, respectively. Those that were retrogradely labelled with CTb and FG are shown in blue, and those labelled only with CTb in red. Only one tdTom cell (in the L2 segment) was not retrogradely labelled, and this is shown in black. Dashed lines represent the dorsal and ventral borders of lamina II. Outline drawings were prepared with XaraXtreme v2 (http://www.xaraxtreme.org/).

To investigate the association between tdTomato-positive cells and ALS neurons, we performed retrograde labelling experiments. Seven *Phox2a::Cre;Rosa26*^*LSL-tdTomato*^ mice received an injection of cholera toxin B subunit (CTb) targeted on the LPb either bilaterally or unilaterally, and two of the mice with unilateral CTb injections also received an injection of Fluorogold into the thalamus on the same side (Table 1, Figs. 1, 2, Supplemental Figs. S2, S3). Transverse sections from these animals were used to determine the proportion of retrogradely labelled lamina I neurons that were tdTomato-positive at two different segmental levels: C7 and L2. This analysis was initially performed on the side contralateral to the injection site for the mice that received unilateral injections (#3–7), while results from both sides were pooled for those mice with bilateral injections (#1, 2). Quantitative data from this part of the study are shown in Table 2, examples of immunostaining in Fig. 2, and plots showing the distribution of cells in Fig. 3 and Supplemental Fig. S4. We identified between 34 and 115 tdTomato-positive lamina I cells in the L2 segments of these mice (mean 6.9 ± 1.2 cells per 60 μm section), and found that the vast majority of these (94–100%) were retrogradely labelled with CTb. Conversely, tdTomato was present in 59% (range 49–68%) of the CTb-labelled cells in lamina I in this segment. In the C7 segment we identified between 32 and 101 tdTomato-positive lamina I cells (mean 9.9 ± 1.5 cells per 60 μm section), and we found two patterns with respect to Phox2a expression. In 4 of the mice (#1, 2, 4 and 6), the great majority (95–99%) of tdTomato cells were CTb-labelled. However, in the other 3 mice (#3, 5, 7) the proportion was considerably lower (72%, 78%, 83%, respectively) even though 94–100% of the tdTomato cells in the L2 segments of these three mice were CTb-labelled (Table 2). The mean proportion of CTb-labelled cells in C7 that contained tdTomato was 54% (range 45–64%), and this did not differ significantly from the corresponding proportions seen in the L2 segment (paired t-test, p = 0.19). We also noted that although retrogradely-labelled lamina I cells were evenly distributed across the mediolateral axis in both the L2 and T6 segments, they were consistently clustered in the middle part of the dorsal horn at C7 (Fig. 2, Supplemental Fig. S4). However, tdTomato-labelled dendrites extended into both medial and lateral parts of the dorsal horn at this level. Since the input to the dorsal horn is somatotopically organised, this suggests that the relationship between soma location and receptive field may differ between cervical enlargement and other spinal regions.

**Table 1:** Injection sites in mice used for anatomical studies.

Experiment	Injection 1			Injection 2		
	Target	Tracer	Volume (nl)	Target	Tracer	Volume (nl)
1	LPb (L)	1% CTb	300	LPb (R)	1% CTb	300
2	LPb (L)	1% CTb	300	LPb (R)	1% CTb	300
3	LPb (L)	1% CTb	300			
4	LPb (L)	1% CTb	300			
5	LPb (L)	1% CTb	300			
6	LPb (L)	1% CTb	300	Thalamus (L)	2% FG	100
7	LPb (L)	1% CTb	300	Thalamus (L)	1% FG	100

**Table 2:** Quantitative data for retrograde tracing experiments involving injection of cholera toxin B (CTb) into the lateral parabrachial area.

Experiment	L2 contra		L2 ipsi		L3 contra		C7 contra		C7 ipsi	
	% Phox2a with CTb	% CTb with Phox2a	% Phox2a with CTb	% CTb with Phox2a	% Phox2a with CTb	% CTb with Phox2a	% Phox2a with CTb	% CTb with Phox2a	% Phox2a with CTb	% CTb with Phox2a
1	98.6 (69/70)	68.3 (69/101)			99.3 (134/135)	69.4 (134/193)	99 (100/101)	54.6 (100/183)		
2	95.7 (110/115)	62.5 (110/176)			98.5 (133/135)	70 (133/190)	94.8 (92/97)	61.3 (92/150)		
3	97.7 (42/43)	60.9 (42/69)	37.2 (16/43)	57.1 (16/28)	100 (60/60)	56.1 (60/107)	71.7 (38/53)	47.5 (38/80)	28.2 (11/39)	25.6 (11/43)
4	98 (50/51)	52.1 (50/96)	2.5 (1/40)	9.1 (1/11)	98 (48/49)	52.2 (48/92)	95.7 (67/70)	52.3 (67/128)	22.8 (13/57)	27.1 (13/48)
5	94.1 (32/34)	57.1 (32/56)	30.6 (11/36)	32.4 (11/34)	98.1 (53/54)	50.5 (53/105)	78.1 (25/32)	45 (25/55)	26.0 (13/50)	41.9 (13/31)
6	98 (50/51)	49 (50/102)	4.4 (2/45)	20.0 (2/10)	100 (33/33)	45.8 (33/72)	98.6 (72/73)	55 (72/131)	28.1 (16/57)	36.4 (16/44)
7	100 (53/53)	62.4 (53/85)	14.5 (8/55)	27.6 (8/29)			83.3 (57/69)	64.2 (57/109)	12.0 (9/75)	21.4 (9/42)

Cells show percentages, with the actual numbers in parentheses. Note that in experiments 1 and 2 CTb was injected bilaterally into the LPb. Data from both sides were pooled for the L2 and C7 segments and this is entered under “contra” in both cases.

For those mice that received unilateral injections of CTb into the LPb, we also analysed the side ipsilateral to the injection for the L2 and C7 sections (Table 2). As expected, we found that a lower proportion of tdTomato cells were retrogradely labelled (mean 15%, range 3–37%). However, we also found that the proportion of retrogradely labelled lamina I cells that were tdTomato-positive was significantly lower than on the contralateral side (mean 30%, range 9–57% for ipsilateral, mean 55%, range 45–64% for contralateral, paired t-test, p < 0.001). Overall, these results are generally consistent with those reported by Roome et al.^7^, since they reported that the *Phox2a::Cre* mouse captured 40–50% of retrogradely labelled lamina I cells on the side contralateral to the injection site, but only ~ 20% of those on the ipsilateral side.

In three of the experiments (#1, 2, 6) we also quantified the proportion of retrogradely labelled LSN neurons that were tdTomato-positive. We counted an average of 89 LSN cells (range 46–134) in the L2 segment and found that 8.5% (range 6.5–11.6%) of these were tdTomato-positive. The corresponding numbers for C7 were 61 cells (27–95), of which 10.7% (7.4–13.1%) contained tdTomato. This indicates that the *Phox2a::Cre* line captures relatively few LSN projection neurons.

### Phox2a lamina I cells and the spinothalamic tract

We then examined the relationship between neurons that were retrogradely labelled from the thalamus, those labelled from the LPb and cells that were tdTomato-positive. In the two mice that also received thalamic injections (#6, #7), Fluorogold was largely restricted to the thalamus and the injection site included parts of the posterior and posterior triangular nuclei in both cases, with extension into the ventral posterolateral (VPL) and ventral posteromedial nuclei in #7 (Supplemental Fig. S3). We quantified Fluorogold-labelled lamina I neurons on the contralateral side in the L2 and C7 transverse sections. The general pattern of labelling was similar to what we had reported in the rat^21^, with relatively few retrogradely labelled lamina I cells in the lumbar region and far more in the cervical cord (Table 3, Figs. 2, 3, Supplemental Fig. S4). In the L2 segment all Fluorogold cells were also CTb-positive, and these accounted for 6.7% (3.5%, 9.8%) of the CTb cells. In the C7 segment 91.7% of Fluorogold cells were CTb (83.3%, 100%), and these corresponded to 72.2% (64.2%, 80.2%) of the cells that were CTb-labelled. In the rat, we had estimated that the proportions of lamina I ALS cells that projected to thalamus were ~ 5% and ~ 45% for lumbar and cervical enlargements, respectively^21^. The present results show that while there are very few spinothalamic lamina I cells in the lumbar region, these are far more numerous in the cervical cord, making up nearly three-quarters of lamina I projection cells. It is possible that incomplete labelling of the VPL nucleus in these experiments contributed to the low number of lamina I spinothalamic cells that we saw in the L2 segment. However, this is unlikely because we have shown in rat that very few lamina I neurons in the lumbar enlargement are retrogradely labelled following tracer injections that fill the VPL, and that most lamina I spinothalamic neurons at both lumbar and cervical levels can be retrogradely labelled from the posterior triangular nucleus^33^.

**Table 3:** Quantitative data for retrograde tracing experiments involving injection of Fluorogold (FG) into the thalamus.

Experiment	L2				C7			
	% Phox2a with FG	% FG with Phox2a	% FG with CTb	% CTb with FG	% Phox2a with FG	% FG with Phox2a	% FG with CTb	% CTb with FG
6	9.8 (5/51)	50 (5/10)	100 (10/10)	9.8 (10/102)	78.1 (57/73)	54.3 (57/105)	100 (105/105)	80.2 (105/131)
7	3.8 (2/53)	66.7 (2/3)	100 (3/3)	3.5 (3/85)	68.1 (47/69)	56 (47/84)	83.3 (70/84)	64.2 (70/109)

Cells show percentages, with the actual numbers in parentheses.

As was the case for cells that were retrogradely labelled with CTb, Fluorogold cells included both Phox2a+ and Phox2a− types. Specifically, in the C7 segment 55.2% (56%, 54.3%) of FG-labelled cells were Phox2a, which is similar to the proportion of CTb-labelled cells with Phox2a in this segment (52%). This confirms the finding of Roome et al.^7^ that Phox2a is not preferentially expressed by those ALS neurons that project to the thalamus.

### Differences in soma size and NK1r expression among Phox2a+ and Phox2a− lamina I projection neurons

**Figure 4:**
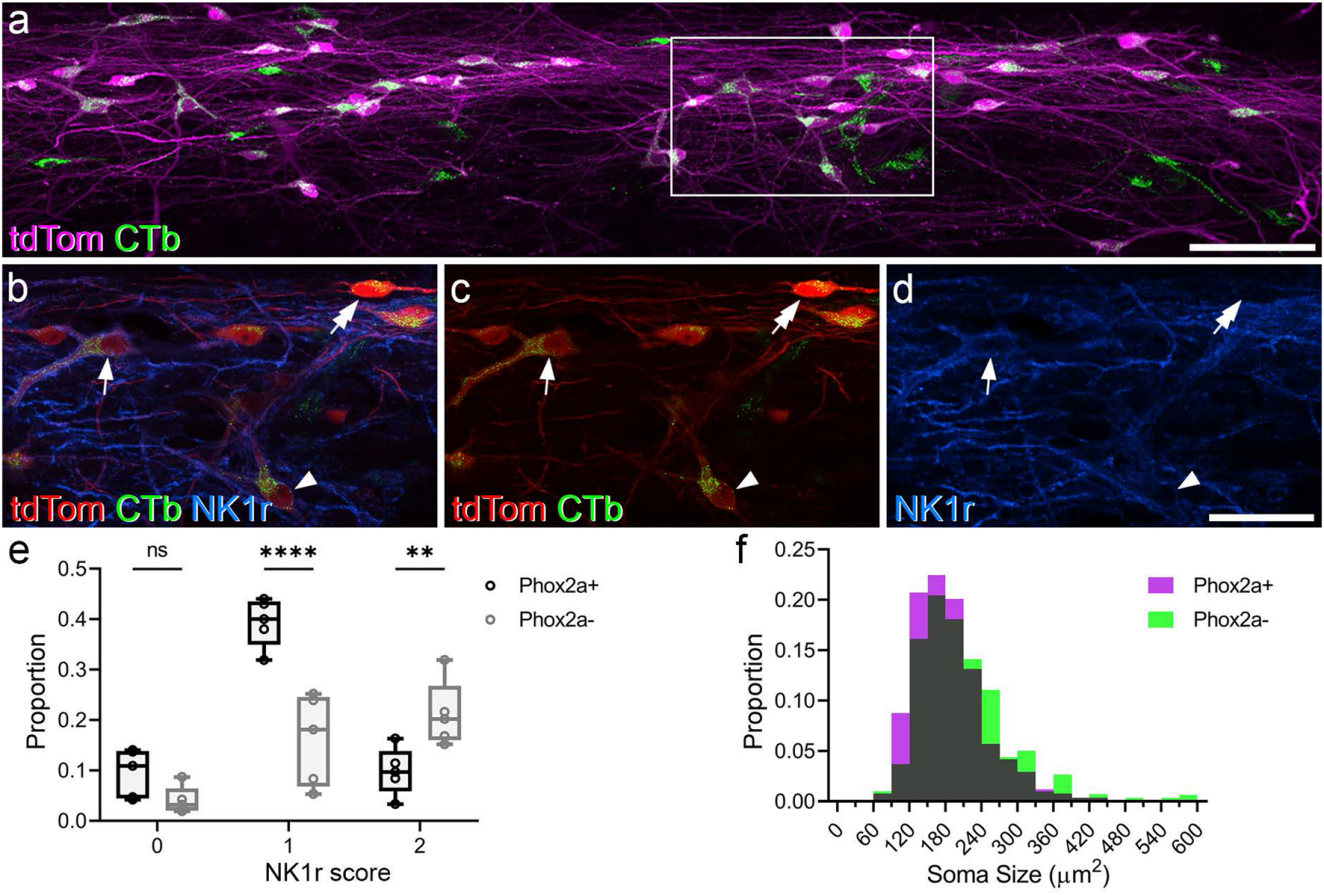
Retrogradely labelled and tdTomato-positive lamina I neurons in horizontal sections from the L3 segment. (**a**) Part of lamina I from experiment #1, in which cholera toxin B subunit (CTb) was injected bilaterally into the lateral parabrachial area. TdTom and CTb are revealed in magenta and green, respectively. Many double-labelled cells are visible, as well as some that are CTb-positive but lack tdTomato. The box shows the area corresponding to (**b**–**d**). (**b**–**d**) Show this region at higher magnification, with NK1r-immunoreactivity in blue, tdTom in red and CTb in green. Several retrogradely labelled tdTom-positive cells are visible in this field. The three marked cells are either strongly NK1r-immunoreactive (arrow), weakly NK1r-immunoreactive (arrowhead) or NK1r-negative (double arrow). The image in (**a**) is projected from a confocal scan through the thickness of the section, while those in (**b**–**d**) are projected from 2 optical sections (1 μm z-separation in each case). Scale bars: (**a**) = 100 μm, (**b**–**d**) = 50 μm. (**e**) Box and whisker plots showing the proportions of Phox2a-positive and -negative cells that were classed as negative (0), weakly-stained (1) or strongly stained (2) for NK1r in experiments #1–4 and #6. Significance is shown with asterisks (**p < 0.01; ****p < 0.0001). (**f**) Frequency histogram showing the soma sizes of retrogradely labelled neuron analysed in experiments #1–#6. Phox2a-positive and negative cells are represented in magenta and green, respectively, and areas of overlap are shown in grey.

We next tested whether tdTomato was preferentially expressed in particular types of lamina I projection neuron, based on cell size or NK1r expression. We reacted horizontal sections from the L3 segments of 6 of the mice (#1–6) with antibody against NeuN to label cell bodies. In 5 of these cases (#1–4, 6), we also detected NK1r immunostaining. Again, the analysis was carried out on the contralateral side for mice that received unilateral injections and on both sides for those with bilateral injections. We identified between 72 and 193 CTb-labelled lamina I neurons in these mice, and these were classified as negative, weakly stained or strongly immunostained for the NK1r (Fig. 4a–d). In this part of the study we found that between 46 and 70% (mean 56%) of the CTb-labelled cells contained tdTomato, while 98–100% of the tdTomato-positive cells were labelled with CTb, which is similar to the pattern seen in the transverse sections from the L2 segments (Table 2).

Overall, we found that 13.5% of CTb-labelled cells were NK1r-negative (range 6.5–19.4%). Cells classed as weak NK1r-positive accounted for 55.6% (45.3–68.2%) of CTb cells, while those defined as strong NK1r-positive made up 31% (18.5–41.7%). The proportion of retrogradely labelled cells that were NK1r-negative did not differ between the Phox2a positive and negative subsets (Fig. 4e). However, a significantly greater proportion of retrogradely labelled cells were weakly immunoreactive for NK1r in the Phox2a positive subset (ANOVA, p < 0.0001, Fig. 4e). The opposite was true for cells that were strongly immunoreactive for NK1r, among which Phox2a-negative cells were significantly overrepresented (ANOVA, p = 0.0016, Fig. 4e).

A frequency histogram of soma sizes for the Phox2a-positive and -negative cells is shown in Fig. 4f. The mean somatic area in Phox2a-positive cells was 186 µm^2^ (SD 58 µm^2^), while that for the Phox2a-negative cells was 209 µm^2^ (SD 77 µm^2^), and this difference was significant (p = 0.002, linear mixed model).

Together, these findings show that the *Phox2a::Cre* mouse line preferentially targets the smaller lamina I ALS neurons, and that the Phox2a cells are less likely to express high levels of the NK1r than the Phox2a-negative cells.

### The relation of Phox2a to other neurochemical markers for projection neurons

**Figure 5:**
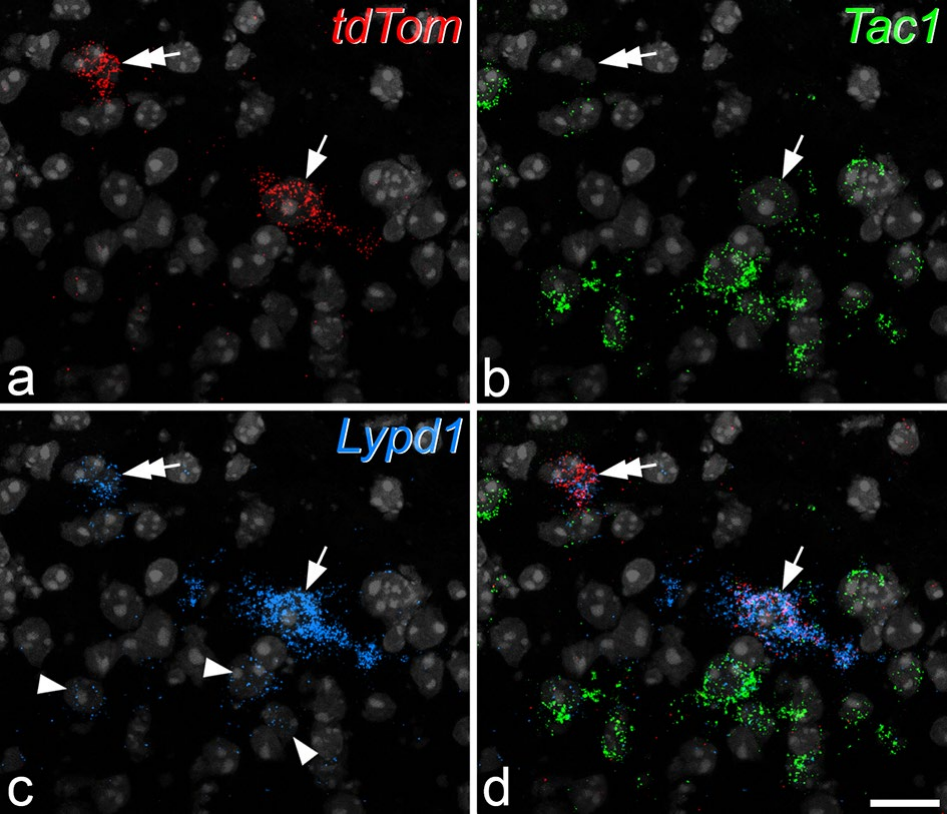
Fluorescence in situ hybridisation with RNAscope in a transverse section from a *Phox2a::Cre;Rosa26*^*LSL-tdTomato*^ mouse. The section has been reacted with probes directed against *tdTom* (red), *Tac1* (green) and *Lypd1* (blue) mRNAs. These are shown separately in (**a**–**c**), and combined in (**d**). The nuclear counterstain NucBlue is shown in grey. Two *tdTom*-positive cells are present in this field. One of these (arrow) is positive for *Tac1*, while the other (double arrow) is not. Several smaller *Tac1*-positive cells are present, and these are likely to be interneurons. Both of the *tdTom*-positive cells are also positive for *Lypd1*. However, many other smaller cells show weak *Lypd1*, and some of these are indicated with arrowheads. Images are projections of confocal optical sections (1 μm z-separation) through the full thickness of the section. Scale = 20 μm.

Previous studies have defined subpopulations of lamina I ALS cells based on differential expression of Tac1, Tacr1 and Gpr83^28,29^. In addition, it has been suggested that Lypd1 represents a reliable marker for lamina I spinoparabrachial neurons, since 85% of these cells were Lypd1-positive^34^. We therefore performed multiple-labelling fluorescent in situ hybridisation on sections from *Phox2a::Cre;Rosa26*^*LSL-tdTomato*^ mice to investigate the pattern of expression of these mRNAs in tdTomato-positive cells. We initially reacted transverse sections from 3 mice with probes against *Tac1*, *Lypd1* and *tdTom* mRNAs. We identified 120 lamina I *tdTom*^+^ cells (35–48 cells per mouse) and found that 40.6% (range 37–44%) of these were positive for *Tac1* mRNA. All but 2 (97–100%) of the *tdTom* cells were strongly positive for *Lypd1* mRNA (Fig. 5). However, *Lypd1* mRNA was also present at lower levels in many other neurons in the superficial dorsal horn, with around 30–40 cells in laminae I-II per 12 μm section having at least 5 transcripts. Since there are around 10 spinoparabrachial lamina I cells per 60 μm section in the mouse midlumbar spinal cord^32^, expression of Lypd1 is apparently not restricted to projection neurons.

**Figure 6:**
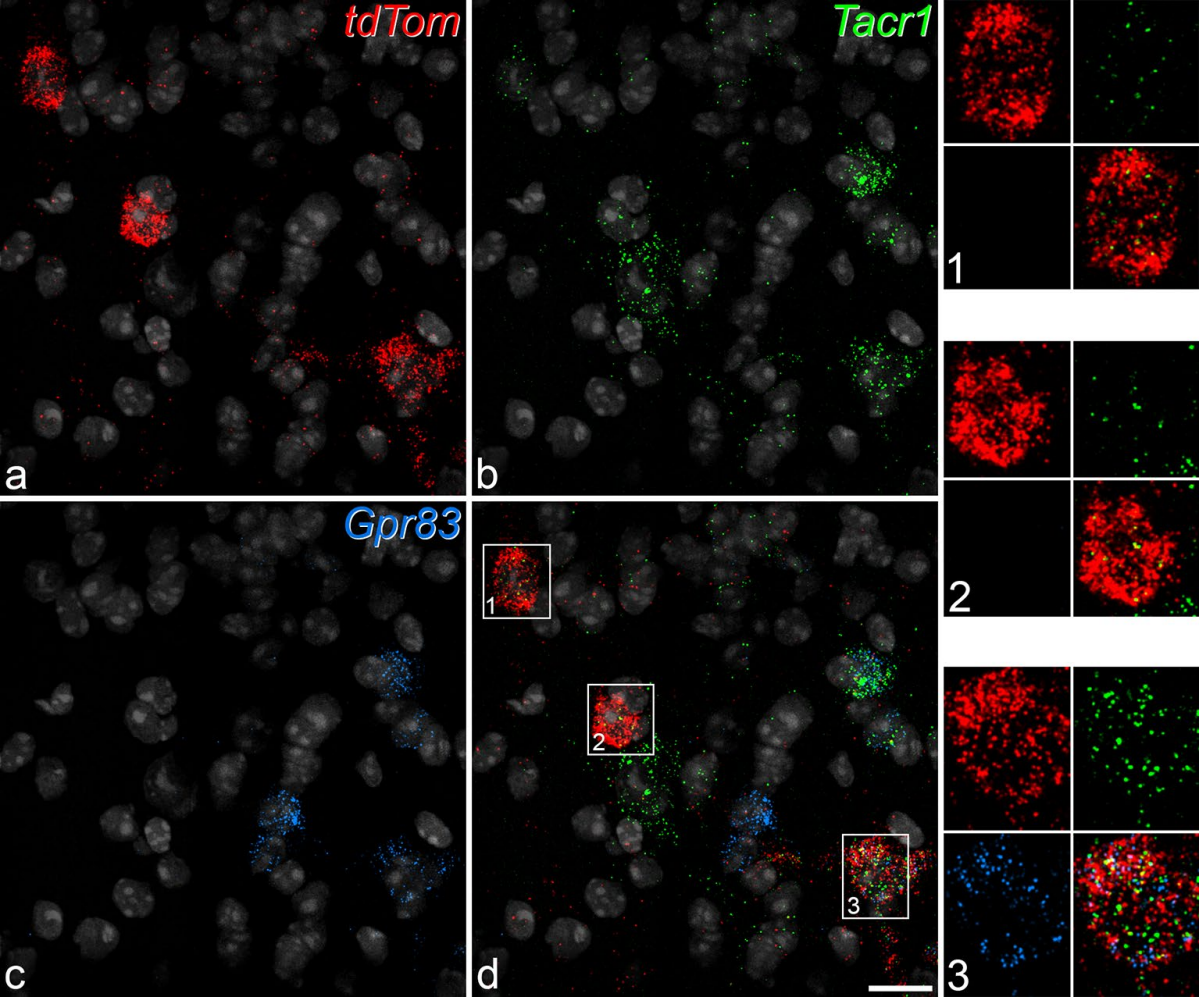
Fluorescence in situ hybridisation with RNAscope in a horizontal section from a *Phox2a::Cre;Rosa26*^*LSL-tdTomato*^ mouse. The section was reacted with probes for *tdTom* (red), *Tacr1* (green) and *Gpr83* (blue) mRNAs, and these are shown separately in (**a**–**c**) and combined in (**d**). The nuclear counterstain NucBlue is shown in grey. Three *tdTom*-positive cells are present (numbered 1–3 in (**d**)). These are illustrated at higher magnification in the insets, and in each of these the top row shows labelling for *tdTom* and *Tacr1* and the bottom row labelling for *Gpr83* together with a merged image. All 3 cells are positive for *Tacr1* (based on the presence of more than 5 transcripts), while cell 3 is positive for *Gpr83*. Note that there are sparse scattered transcripts for *tdTom*, which are also seen in *Rosa26*^*LSL-tdTomato*^ mice, and presumably result from a low-level “leak” of tdTomato expression. However, these can easily be distinguished from the labelling in the putative Phox2a-positive lamina I neurons. Images are projections of confocal optical sections (1 μm z-separation) through the full thickness of the section. Scale = 20 μm.

**Figure 7:**
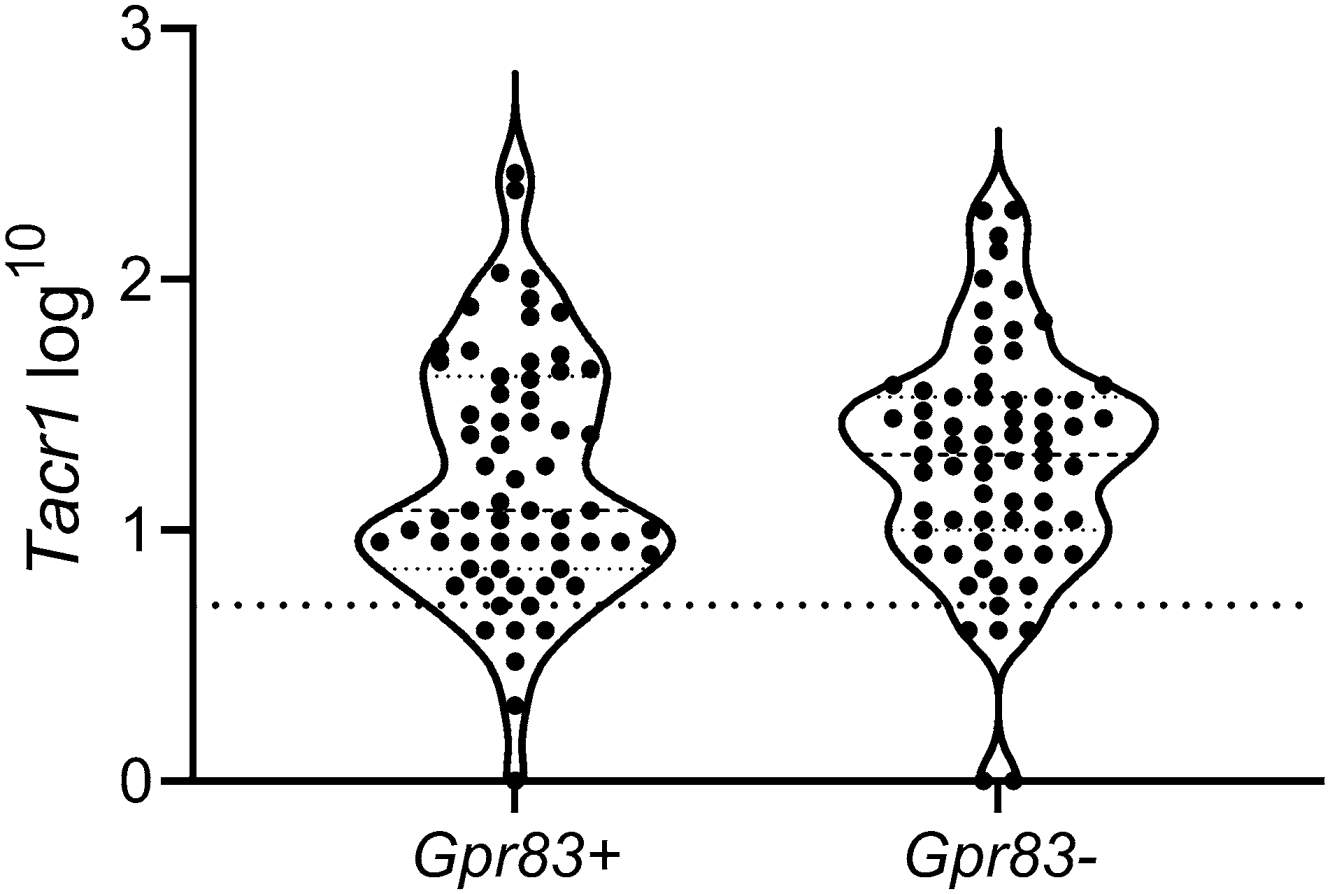
Violin plot of *Tacr1* transcript number in *Gpr83*-positive and *Gpr83*-negative *Phox2a* neurons. The dotted line represents 5 transcripts per cell (i.e. the threshold for defining positivity). Lines within the violins represent the median and inter-quartile range.

We then used horizontal sections through lamina I to examine the expression of Tacr1 and Gpr83 in tdTom-positive cells. In this part of the study, we found 131 *tdTom*^+^ cells in the 3 mice (37–51 cells per mouse), of which 90% (range 86–95%) were *Tacr1*^+^ and 48% (range 43–51%) were *Gpr83*^+^ (Fig. 6). There was no correlation between the number of transcripts for *Tacr1* and *Gpr83* in individual cells (Pearson’s r = 0.073, p = 0.408). We then plotted *Tacr1* transcript numbers for both *Gpr83*^+^ and *Gpr83*^−^ cells. We found that while there was a trend towards lower *Tacr1* transcript numbers among *Gpr83*^+^ cells, this was not significant (t-test, p = 0.365; Fig. 7). This suggests that for the Phox2a^+^ subset of lamina I ALS neurons, the expression of NK1r does not differ significantly between the Gpr83-positive and negative cells, at least at the mRNA level, despite the apparent separation between Tacr1 and Gpr83 populations^28^.

### Responses to noxious heat

**Figure 8:**
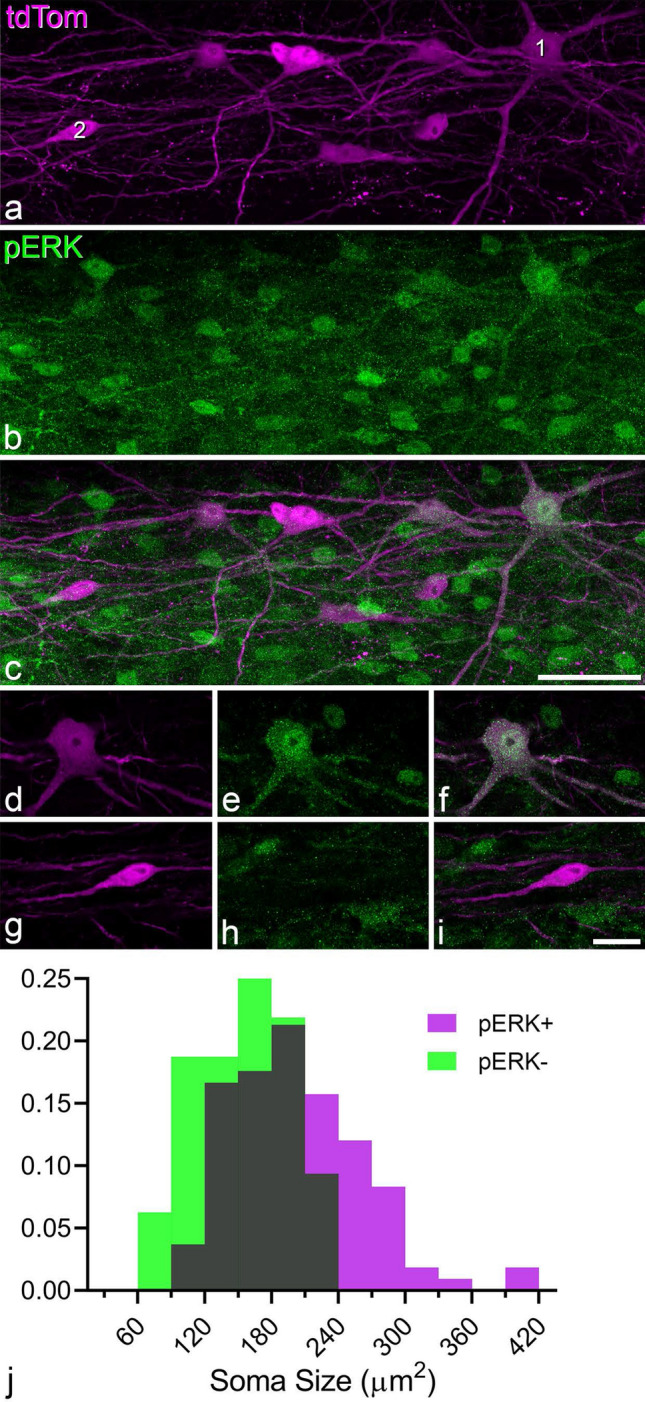
Phosphorylation of extracellular signal-regulated kinases (ERK) in response to noxious heat stimulation. (**a**,**b**) part of a horizontal section through the ipsilateral dorsal horn from a *Phox2a::Cre;Rosa26*^*LSL-tdTomato*^ mouse that had received a noxious heat stimulus to one hind paw. The tissue was scanned to reveal tdTomato (tdTom, magenta) and phosphorylated ERK (pERK, green), respectively and the images are projections of 10 optical sections at 2 μm z-spacing. Two cells are numbered in (**a**). (**c**) A merged image shows that the Phox2a neurons are in a region that contains numerous pERK-positive cells. (**d**–**i**) single optical sections through the two Phox2a cells numbered 1 and 2 in (**c**). Cell 1 is pERK-positive and cell 2 is pERK-negative. Scale bars: (**a**–**c**) = 50 μm, (**d**–**i**) = 20 μm. (**j**) A frequency histogram showing the sizes of the Phox2a-positive cells that were pERK+ and pERK−, expressed as a proportion of each population.

Most lamina I ALS neurons are activated by noxious somatic stimuli and consequently should show phosphorylation of extracellular signal regulated kinases (ERK)^35^. We therefore tested whether Phox2a-positive cells had detectable levels of phosphorylated ERK (pERK) after noxious heat stimulation of the hindlimb (Fig. 8a–i). We analysed 140 cells from three animals (36–60 cells per mouse) and found that 108 (mean 78%, range 71–86%) of these were positive for pERK. We also measured the somatic area of these Phox2a-positive cells and found that larger cells were more likely to phosphorylate ERK (Fig. 8j; Linear mixed model, p < 0.001).

### Dendritic spines and excitatory synapses on Phox2a cells in lamina I

**Figure 9:**
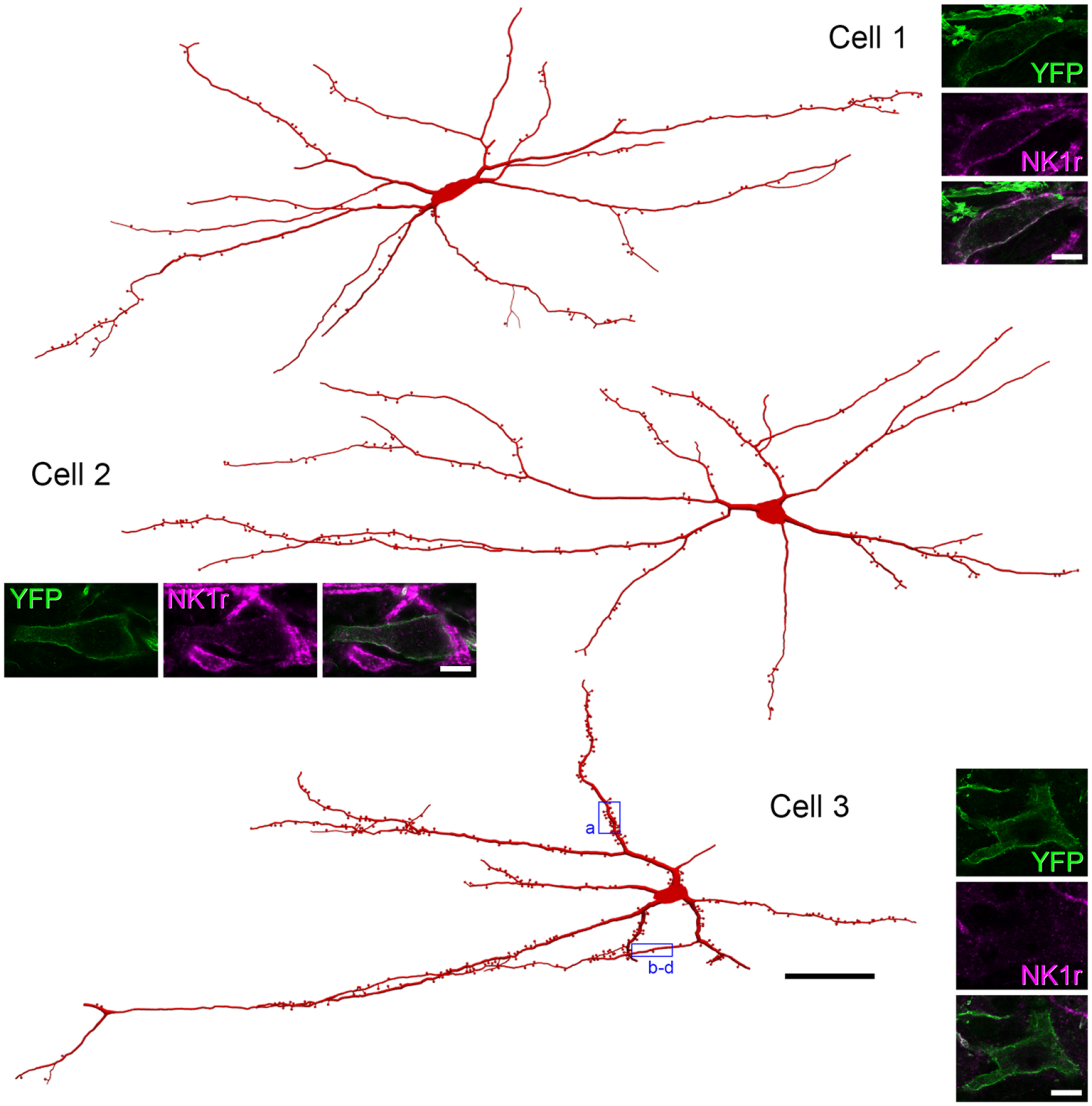
Neurolucida reconstructions of Phox2a cells from horizontal sections of *Phox2a::Cre;Rosa26*^*LSL-ChR2-EYFP*^ mice. The drawings show the cell bodies and dendritic trees of 3 YFP-positive cells. Note that although the locations of all dendritic spines are shown, these are represented in a standardised way that does not indicate spine shape. Insets show confocal scans through the cell body of each neuron. YFP is shown in green and NK1r-immunoreactivity in magenta. Cell 1 was classed as strongly NK1r-immunoreactive, Cell 2 as weakly NK1r-immunoreactive and Cell 3 as NK1r-negative. Boxes over Cell 3 show regions illustrated in Fig. 11. Scale bars: main figure = 100 μm; insets = 10 μm. Neurons were reconstructed with Neurolucida v2020.1.3 (https://www.mbfbioscience.com/).

**Figure 10:**
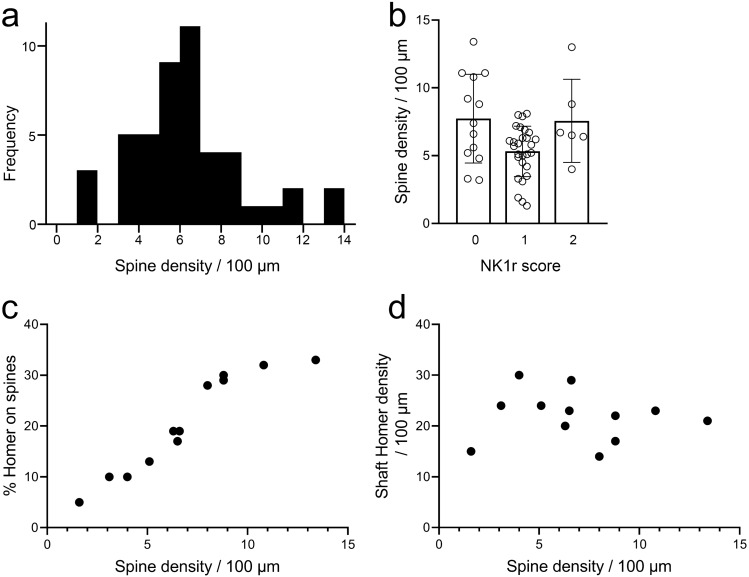
Quantitative analysis of dendritic spines and Homer puncta. (**a**) Frequency histogram showing the range densities on the 47 neurons analysed from the 3 *Phox2a::Cre;Rosa26*^*LSL ChR2-EYFP*^ mice. (**b**) Spine densities for cells classified as strongly NK1r-immunoreactive (2), weakly immunoreactive (1) or non-immunoreactive (0). There was no significant difference between the three classes. Mean and SD are indicated. (**c**) There was a strong correlation between the dendritic spine density and the proportion of Homer puncta that were located on spines for the 12 cells in which Homer was analysed. (**d**) There was no correlation between spine density and the density of Homer puncta that were located on dendritic shafts for these cells.

Little is known about the distribution of dendritic spines on lamina I ALS neurons, or about the proportion of the excitatory synapses onto these cells that are located on spines versus dendritic shafts. To investigate these features, we crossed *Phox2a::Cre* mice with the *Rosa26*^*LSL-ChR2-EYFP*^ line. This generated *Phox2a::Cre;Rosa26*^*LSL-ChR2-YFP*^ mice, in which Phox2a-expressing cells were labelled with a fusion protein consisting of channelrhodopsin and yellow fluorescent protein (YFP) that was targeted to the plasma membrane. We reconstructed the dendritic trees of 47 lamina I Phox2a-positive cells from 3 mice (14–17 cells from each mouse) and plotted the distribution of their dendritic spines (Fig. 9). All of these cells possessed dendritic spines, although the density varied considerably, ranging from 1.3 to 13.4 (mean 6.3 ± 2.7 SD) spines per 100 μm of dendritic length (Fig. 10a). Neurons were assessed for NK1r immunoreactivity, and as above, they were assigned to 3 groups: negative (n = 13), weakly positive (n = 28) and strongly positive (n = 6). The spine density per 100 μm did not vary significantly between these groups (negative: 7.7 ± 3.3; weakly positive: 5.2 ± 2.4; strongly positive: 6.6 ± 2.3; p = 0.170, Linear mixed model) (Fig. 10b).

**Figure 11:**
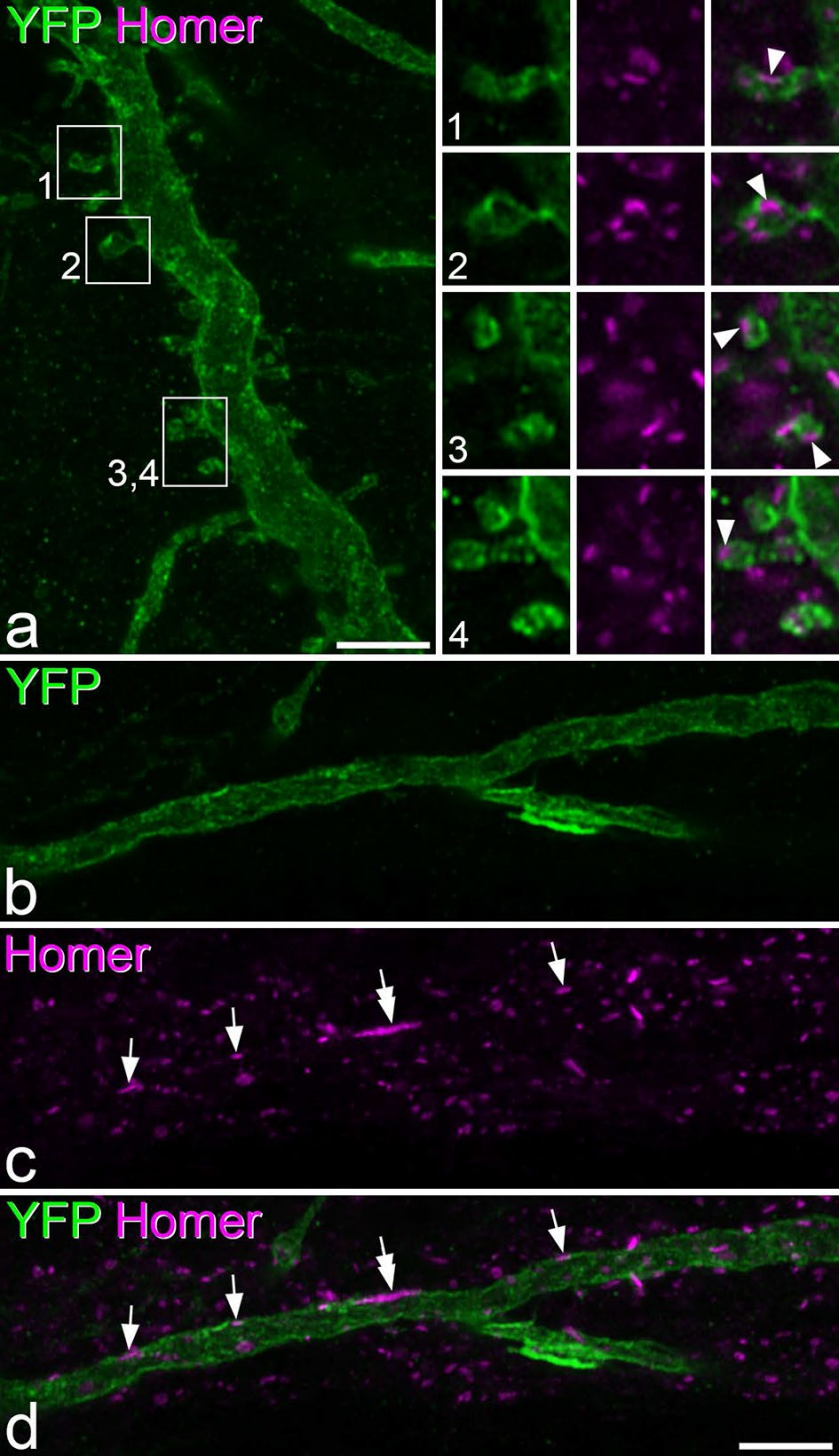
Homer puncta on the dendritic shafts and spines of YFP-positive lamina I cells in *Phox2a::Cre;Rosa26*^*LSL- ChR2-EYFP*^ mice. (**a**) A region of dendrite from Cell 3 (shown in Fig. 9) that has numerous dendritic spines. The insets show higher magnification views of these spines. The tissue has been reacted to reveal YFP (green) and Homer (magenta). Homer puncta can be seen in each of the dendritic spines (arrowheads) in the insets. (**b**–**d**) Part of a dendritic shaft from the same cell. This region has few dendritic spines, but there are several Homer puncta in the membrane (arrows). One of these (double arrow) is particularly large (approximately 3 μm long). Confocal images are projections of 11 optical sections (main part of (**a**) and 4 optical sections (**b**–**d**) all at 0.3 μm z-separation. Insets in (**a**) are all single optical sections. Scale bars: (**a,b**–**d**) = 5 μm.

We analysed the pattern of Homer expression on 12 of these cells (6 each from 2 of the mice), and these were selected to include cells with a range of spine densities (mean 6.9, range 1.6–13.4 spines per 100 μm dendrite length). Homer puncta were detected on both dendritic spines and shafts, and could be clearly recognised by their location within the membrane, which was outlined by YFP (Fig. 11). Homer puncta were visible on the majority of dendritic spines (72 ± 13%, n = 12 cells), but these were invariably outnumbered by Homer puncta on dendritic shafts, such that the proportion of Homer puncta that were located on dendritic spines varied from 5 to 33% (mean 21%). Although there was a strong correlation between the proportion of Homer puncta that were on dendritic spines and spine density (Pearson correlation coefficient, r = 0.952, p < 0.001), there was no correlation between spine density and the density of Homer puncta on shafts (r =  − 0.09, p = 0.78) (Fig. 10c,d). This suggests that the pattern of synaptic input onto the dendritic shafts of these projection neurons is not related to the density of dendritic spines.

Although most of the Homer puncta on dendritic shafts of these cells were less than 1 μm in length, we found that some were considerably larger (> 1.5 μm), consistent with our findings for projection neurons in the rat dorsal horn^36,37^. These “giant” Homer puncta were observed on 10 of the 12 cells, including cells that lacked NK1r and cells with weak and strong NK1r expression. The number of giant Homer puncta varied from 3 to 46 per cell, and they accounted for between 1 and 10% (mean 4%) of all Homer puncta on the dendritic shafts.

## Discussion

Our main findings are: (1) that the *Phox2a::Cre* line captures 50–60% of lamina I spinoparabrachial cells, and that expression is essentially restricted to projection neurons in this lamina; (2) that the pattern of expression is not random, since larger cells and those with strong NK1r-immunoreactivity are less likely to be Phox2a-positive; (3) that although all of the Phox2a cells in lamina I possessed dendritic spines, these accounted for well under half of the Homer puncta on individual cells, and spine synapses are therefore likely to represent a minor component of the excitatory input to these cells.

### Phox2a and the types of projection neuron captured

Roome et al. had concluded that all Phox2a-positive cells in lamina I were projection neurons, since in some cases they achieved 100% labelling of these cells following injection of tracers into the LPb and thalamus^7^. We consistently labelled ~ 95–100% of lumbar Phox2a lamina I cells on the side contralateral to LPb injection, and in 4 out of 7 cases we obtained a similar result in the cervical enlargement. The small numbers of Phox2a cells that were not retrogradely labelled might correspond to ALS neurons that only project to the ipsilateral side of the brain, or else cells that project to other brain regions and do not have axons passing through the parabrachial area. Our findings therefore strongly suggest that Phox2a expression in lamina I is restricted to projection neurons. We had previously shown in the rat that virtually all lamina I projection neurons could be detected following a single injection of retrograde tracer into the LPb^19–21^. The present results confirm that this is also the case in the mouse, at least for those projection cells that are Phox2a-positive. Although it is thought that the input from lamina I ALS cells to the LPb is not somatotopically organised^28^, we found that in 3 of the experiments (#3, 5, 7) a somewhat lower proportion (61–83%) of the Phox2a lamina I cells in the C7 segment were retrogradely labelled, despite nearly complete labelling of cells in the L2 and L3 segments contralateral to the injection. Since 95–100% of the lamina I Phox2a cells in the C7 segment were retrogradely labelled in the other 4 experiments, it is likely that all of the Phox2a lamina I cells at this segmental level are projection neurons. The most likely explanation for the lower proportion of retrogradely labelled cells in experiments #3, 5 and 7 is that at least some cervical lamina I neurons project to distinct parts of the lateral parabrachial complex that were not included in the CTb injections in these experiments.

Although most retrogradely labelled lamina I neurons are located contralateral to LPb injections, some are found on the ipsilateral side. We had previously injected two different tracers into the right and left LPb in the rat, and shown that nearly all of the lamina I cells labelled from ipsilateral LPb were also labelled from the contralateral side, indicating that they projected bilaterally^19^. These cells were found to account for ~ 30% of lamina I ALS neurons. Our finding that virtually all Phox2a cells contralateral to LPb injections were CTb-labelled indicates that this pattern also applies to Phox2a ALS lamina I neurons in the mouse, since if there had been significant numbers of cells that only projected ipsilaterally, these would not have been labelled with CTb injections into the contralateral LPb.

Interestingly, we found that a significantly lower proportion of retrogradely labelled lumbar lamina I cells on the side ipsilateral to the LPb injection were Phox2a-positive compared to those on the contralateral side, and a similar result was obtained by Roome et al. for cervical lamina I cells^7^. This indicates that bilaterally projecting cells are less likely to be Phox2a-positive than those that only project to the contralateral side, and provides further evidence that these represent distinct populations. This is consistent with the recent finding that spinoparabrachial cells with ipsilaterally-projecting axons have a specific role in mediating nocifensive behaviour^38^. Because the CTb-labelled cells on the side contralateral to the LPb injection site will include both bilaterally-projecting and contralaterally-projecting cells, it is likely that for the cells that only project contralaterally the proportion that are Phox2a-positive will be higher than the ~ 55–60% that we find for all CTb-labelled cells.

We used the L2 segment for quantitative analysis of the proportion of Phox2a cells that were retrogradely labelled, and the proportion of retrogradely labelled cells that were Phox2a. Data obtained from this part of the study allow us to compare the numbers of lamina I ALS cells in this segment with values that we have previously reported for the L4 segment^32^. The mean number of retrogradely labelled lamina I cells per 60 μm seen in L2 was 6.9, while the corresponding value for L4 found by Cameron et al.^32^ was 9.9 cells per 60 μm. The mediolateral width of lamina I in the L2 segment is ~ 82% of the corresponding value for L4 (mean 610 μm for L4, compared with 499 μm for L2, MG-M and AJT unpublished observations). However, the density of lamina I ALS cells is ~ 70% of that seen in L4 (6.9 cells rather than 9.9 per 60 μm). This suggests that the packing density of ALS neurons in lamina I is somewhat lower in the L2 segment.

### The relation of Phox2a expression to previously defined populations of lamina I ALS neurons

Lamina I spinoparabrachial neurons are functionally heterogeneous, and it is therefore important to define distinct populations among these cells. Huang et al.^29^ reported that “Tac1 lineage” cells projected to medial thalamus, and were involved in coping behaviours associated with sustained pain. We had previously estimated that ~ 40% of lamina I spinoparabrachial neurons were Tac1-positive, based on Cre expression in a Tac1^Cre^ mouse^39^. Since we find that 40% of the Phox2a cells in lamina I contain *Tac1* mRNA, it appears that Phox2a expression does not discriminate between Tac1-positive and-negative ALS neurons.

Choi et al.^28^ defined two major subsets of lamina I ALS cells by using two different mouse lines: *Tacr1*^*CreERT2*^ and *Gpr83*^*CreERT2*^. They showed that between them, these lines captured around 85% of the projection neurons, with relatively little overlap, and that the two populations were functionally distinct. Among other differences, the Tacr1 and Gpr83 cells responded preferentially to noxious thermal and mechanical stimuli, respectively. The *Gpr83*^*CreERT2*^ cells accounted for 50% of the lamina I spinoparabrachial population, and this is similar to the proportion of Phox2a cells that were *Gpr83*-positive. However, fewer than 60% of the cells were captured in the *Tacr1*^*CreERT2*^ line, whereas we find that nearly 90% of all retrogradely labelled cells were NK1r-immunoreactive, similar to our previous results in both mouse and rat^9,19,32^. Consistent with this, we detected *Tacr1* mRNA in 90% of the Phox2a cells. Surprisingly, in the light of the findings of Choi et al., we saw no significant difference in *Tacr1* transcript numbers between Gpr83-positive and -negative cells. Taken together, these findings suggest that the *Tacr1*^*CreERT2*^ line used by Choi et al. captured only a subset of those cells that express the NK1r, and that both Tacr1- and Gpr83-expressing projection neurons are included among the Phox2a cells. We did find clear differences in terms of NK1r expression level and also soma size, since cells that were relatively small and those with weak NK1r immunostaining were more likely to be Phox2a-positive.

The great majority of lamina I spinoparabrachial cells are activated by noxious heat, and in many cases also by noxious mechanical stimuli^22,23^. However, there is also a well-defined population known as cool cells that respond exclusively to cold^16,25,26,40^. Hachisuka et al. recently identified these cool cells in a “semi-intact” ex vivo preparation, and reported that they had small soma sizes relative to other spinoparabrachial cells, and responded weakly, if at all, to bath-applied substance P. It is therefore likely that they would be classed as weak or negative in terms of NK1r expression. These findings suggest that the cold cells may be among those that are Phox2a-positive. Consistent with this, we found that those Phox2a cells that did not show pERK following noxious heat stimulation had significantly smaller soma areas than the cells that were pERK-positive (and therefore activated by noxious heat). It is possible that lack of pERK may have resulted from the cells having receptive fields that were not stimulated, but this is unlikely, as we applied the stimulus to a large area of skin, and the cells sampled were all within the zone of pERK-immunoreactive neurons. These findings are consistent with the idea that cool cells are among the Phox2a population. However, electrophysiological or imaging studies will be needed to confirm this prediction.

### Dendritic spines and excitatory synaptic inputs

The membranous expression of the YFP-channelrhodopsin fusion protein in the *Phox2a::Cre;Rosa26*^*LSL-ChR2-YFP*^ mice allowed clear visualisation of dendritic spines, and also of Homer puncta in both dendritic spines and shafts. There have apparently been few reports of dendritic spines on lamina I projection neurons, although Antal et al.^41^ examined 5 lamina I projection neurons and found a mean spine density of 5/100 μm, which is consistent with the present result. We found that all of the cells possessed dendritic spines, although the mean density (6.3 spines/100 μm of dendrite) was considerably lower than that on some other neurons in the superficial dorsal horn, since we have reported values of 17–19 spines/100 μm for excitatory interneurons with substance P or that express green fluorescent protein (GFP) in the transgenic *GRP::eGFP* line^42^. In addition, we were able to detect Homer puncta on the majority of spines, indicating the presence of excitatory synapses^43^. Nonetheless, these represented only around one-fifth of the Homer puncta that were present on the cells, indicating that the majority of excitatory synapses on these cells are located on dendritic shafts. In addition, some of the Homer puncta on shafts were very large (> 1.5 μm in length). We have previously identified these giant Homer puncta on both lamina I and lamina III projection neurons in the rat, and have shown that those on the lamina I cells were often associated with boutons that were immunoreactive for calcitonin gene-related peptide, suggesting that they represent excitatory synapses from peptidergic nociceptors^36,37^.

Long-term potentiation (LTP) is a form of synaptic plasticity that involves enlargement of dendritic spines and their associated synapses. Several studies have reported an increase in the density and size of dendritic spines on unidentified populations of dorsal horn neurons in both neuropathic and inflammatory pain states^44–49^. These studies have also shown that preventing spine changes, by interfering with the function of the Rho GTPase Rac1, reduces the pain state, strongly suggesting that spine plasticity contributes to pathological pain. In addition, LTP of C fibre inputs to lamina I projection neurons has been demonstrated following conditioning stimulation of these afferents^50,51^. Although dendritic spines account for the minority of excitatory synapses on the lamina I projection cells, they are likely to play a key role in synaptic plasticity involving these cells^50,51^. It will therefore be important to assess changes in the density and morphology of dendritic spines in models of neuropathic and inflammatory pain, and to look for evidence of an increase in the associated Homer puncta, which would provide direct evidence of synaptic enlargement.

## Methods

### Animals

All experiments were approved by the Ethical Review Process Applications Panel of the University of Glasgow, and were performed in accordance with the European Community directive 86/609/EC and the UK Animals (Scientific Procedures) Act 1986. The study was carried out in compliance with the ARRIVE guidelines.

We used the BAC transgenic *Phox2a::Cre* mouse line in which Cre recombinase is expressed under control of the Phox2a promoter^7^. This was crossed with different reporter lines: *Rosa26*^*LSL-tdTomato*^ (Ai9 or Ai14) and *Rosa26*^*LSL-hChR2-YFP*^ (Ai32), in which Cre-mediated excision of a STOP cassette drives expression of either tdTomato (Ai9, Ai14), or a fusion protein consisting of channelrhodopsin and yellow fluorescent protein (YFP; Ai32).

*Phox2a::Cre;Rosa26*^*LSL-tdTomato*^ mice were used in most parts of the study as the tdTomato clearly labelled cell bodies, dendrites and axons of cells that expressed Cre. However, for the analysis of dendritic spines and Homer puncta, we used the *Phox2a::Cre;Rosa26*^*LSL-hChR2-YFP*^ cross, as this labelled the plasma membrane (due to targeting of the hChR2-YFP fusion protein). The distribution of labelled cells within the spinal cord was very similar in both crosses.

Tissue for immunohistochemistry was obtained from mice that had undergone perfusion fixation. These animals were deeply anaesthetised (pentobarbitone, 20 mg i.p.) and perfused through the left cardiac ventricle with fixative containing 4% freshly depolymerised formaldehyde in phosphate buffer. Spinal cord tissue was removed and post-fixed for 2 h.

### General features of immunohistochemistry and confocal microscopy for spinal cord sections

Spinal cord tissue was cut into 60 μm thick section in either the transverse or horizontal plane with a vibrating blade microtome (Leica VT1200 or VT1000). Sections were processed for multiple-labelling immunofluorescence, as described previously^52–54^. They were incubated at 4 °C in mixtures of primary antibodies (see Table S1) for 3 days and then in species-specific secondary antibodies for 1 day. Antibodies were diluted in PBS containing 0.3 M NaCl, 0.3% Triton-X100 and 5% normal donkey serum. Secondary antibodies (all from Jackson Immunoresearch, West Grove, PA, USA) were all raised in donkey and were conjugated to Alexa488, Alexa647, Rhodamine Red, biotin or horseradish peroxidase (HRP). All secondary antibodies were diluted 1:500 apart from those conjugated to Rhodamine Red (1:100) or HRP (1:200). Biotinylated secondary antibodies were revealed with avidin conjugated to Pacific Blue, while HRP-labelled secondary antibodies were used for tyramide signal amplification (TSA) amplification with a TSA kit (Cyanine 5 NEL 705A001 or tetramethylrhodamine NEL702001; Perkin Elmer Life Sciences, Boston, MA, USA). Following immunoreactions, sections were mounted in anti-fade medium and stored at − 20 °C. They were scanned with Zeiss 710 LSM (Argon multi-line, 405 nm diode, 561 nm solid state and 633 nm HeNe lasers) or Zeiss 900 Airyscan (405, 488, 561, 640 nm diode lasers) confocal microscopes, using 40× or 63× oil-immersion objectives (numerical apertures of 1.3 and 1.4, respectively). In all cases, the aperture was set to 1 Airy unit or less. All analyses were performed with Neurolucida for Confocal software (MBF Bioscience, Williston, VT, USA).

### Retrograde tracing experiments

Seven *Phox2a::Cre;Rosa26*^*LSL-tdTomato*^ mice of either sex (22–32 g) received injections of CTb into the lateral parabrachial area (unilaterally or bilaterally) and in two cases Fluorogold was injected into the thalamus. Details of the injection sites, as well as volumes and concentrations, are provided in Table 1. Mice were anaesthetised with isoflurane and placed in a stereotaxic frame, after which anaesthetic was administered through a mask attached to the frame. Burr holes were made through the skull, and tracers were injected through glass micropipettes, which were left in place for 5 min to minimise leakage back up the track. Animals received peri-operative analgesia (buprenorphine 0.3 mg/kg and carprofen 5 mg/kg) and survived for 3 days after surgery. They were then re-anaesthetised with pentobarbitone (20 mg i.p.) and perfused with fixative as described above. Transverse 100 μm thick sections were cut through the brain to include the extent of the injection site. Those through the parabrachial region were reacted to reveal CTb by incubating for 3 days in anti-CTb (Table S1), followed by an immunoperoxidase reaction^32^. These sections were photographed with a Nikon Optiphot-2 microscope using bright-field or fluorescence optics.

Transverse spinal cord sections from the C7 and L2 segments were immunoreacted to reveal CTb, tdTomato (chicken anti-mCherry antibody), NeuN (mouse antibody) and (for experiments #6 and 7) Fluorogold. For each of the 7 experiments, confocal scans were obtained through the full z-depth of both dorsal horns with 2 μm z-separation (between 9 and 15 sections for L2 and between 6 and 11 for C7). Retrogradely labelled neurons in lamina I were identified and plotted onto outlines of the dorsal horn, using Neurolucida. The presence or absence of tdTom for each of these cells was then recorded, and a search made for any tdTom cells that lacked retrograde tracer. Cells were only included if at least part of the nucleus was contained within the section.

Horizontal sections from the L3 segments were reacted to reveal CTb, tdTomato, NK1r and NeuN (guinea pig antibody). Sections were scanned through the full depth with a z-step of 1 μm. CTb-labelled cells for which the entire soma was present in the section were identified and the soma size (cross-sectional area) was measured by drawing an outline of the cell body^32,55^, based on the pattern of CTb and NeuN immunoreactivity. The intensity of NK1r staining varies considerably among lamina I projection neurons^19,21,55^. However, because of variability in the staining intensity at different depths of the section, and in some cases on cell bodies and dendrites of the same neuron^19^, it would be very difficult to use an objective measure of NK1r staining intensity^55^. We therefore defined cells as either non-immureactive, weakly immunoreactive or strongly immunoreactive for NK1r. The scoring for each cell was agreed by two observers, based on comparison with nearby cells in the same section. To avoid the possibility of bias, the scoring for NK1r was performed before the tdTomato was viewed. The channel corresponding to tdTomato was then examined and cells were classified as tdTom-positive or negative.

### Fluorescent in situ hybridisation

Multiple-labelling fluorescent in situ hybridisation was performed with RNAscope probes and RNAscope fluorescent multiplex reagent kit 320850 (ACD BioTechne; Newark, CA 94560). Fresh frozen lumbar spinal cords obtained from 4 *Phox2a::Cre;Rosa26*^*LSL-tdTomato*^ mice were embedded in OCT mounting medium and cut into 12 μm thick transverse or horizontal sections with a cryostat (Leica CM1950; Leica; Milton Keynes, UK). These were mounted non-sequentially (such that sections on the same slide were at least 4 apart) onto SuperFrost Plus slides (48311-703; VWR; Lutterworth, UK) and air dried. Reactions were carried out according to the manufacturer's recommended protocol. The probes used in this study, and the proteins/peptides that they correspond to, are listed in Table S2. Sections from 3 mice were incubated in the following probe combinations: (1) tdTom, Tac1, Lypd1; (2) tdTom, Tacr1, Gpr83. Sections were mounted with Prolong-Glass anti-fade medium with NucBlue (Hoechst 33342) (ThermoFisher Scientific, Paisley, UK). Positive and negative control probes were also tested on other sections (Table S2). Sections were scanned through the full thickness with a 1 μm z-step.

Analysis of the relationship between TdTomato, Tac1, and Lypd1 was performed manually in Neurolucida (MBF Bioscience, Williston, VT, USA). The entire z-stack was examined, and transcripts belonging to a cell were judged as those distributed either within the nucleus or immediately adjacent to it. Cells were judged as positive for a given mRNA if 5 or more transcripts were present. Those cells that were TdTomato positive were marked prior to viewing the other channels. Analysis of the relationship between TdTomato, Gpr83 and Tacr1 was conducted in Qupath^56^ on a single representative optical section from the centre of the image stack. TdTomato positive cells were identified and the boundary of the soma of each was drawn manually. The subcellular objects feature was used to determine the number of mRNA puncta for each channel per cell.

### Noxious heat stimulation

Three *Phox2a::Cre;Rosa26*^*LSL-tdTomato*^ mice (either sex, 22–28 g) were used to assess ERK phosphorylation in Phox2-positive cells in response to noxious heat. The animals were anaesthetised with urethane (50–60 mg, i.p.) and the right hindlimb (up to the level of the knee joint) was immersed in water at 52 °C for 15 s. After 5 min they were perfused with fixative, as described above. Horizontal sections were cut through the L4 segments, and these were immunoreacted to reveal pERK and tdTomato. Sections containing lamina I were scanned through the full thickness (2 μm z-separation). They were analysed with Neurolucida, and the region that contained pERK-positive cells was outlined. TdTomato-positive lamina I cells within this region were identified and expression of pERK was recorded in each case.

### Analysis of dendritic spines and Homer puncta

Horizontal sections from the L3 segments from 3 *Phox2a::Cre;Rosa26*^*LSL-ChR2-YFP*^ mice were reacted to with antibodies against GFP, NK1r, Homer and VGLUT2, and sections were scanned with a z-step of 0.5 μm. Note that the GFP antibody also detects YFP. Between 14 and 17 cells were selected in each animal for reconstruction (total 47 cells). Cells were selected based on the ability to differentiate their cell bodies and dendrites from those of surrounding cells. The cells were reconstructed with Neurolucida and locations of dendritic spines were added to the drawings. Presence or absence of NK1r immunoreactivity was recorded for each reconstructed cell, and NK1r immunostaining was scored as strong or weak (as above). The analysis of Homer puncta was performed on 6 cells each from two of the mice. These were selected to represent cells with a wide range of spine densities, and the selection was made before Homer distribution on individual cells was visualised. All Homer puncta identified were plotted onto drawings of the selected cells, and those with lengths exceeding 1.5 μm were noted.

### Characterisation of antibodies

Specificity of the antibodies against CTb, Fluorogold, green fluorescent protein (GFP) and mCherry were demonstrated by the lack of staining in regions that lacked retrogradely-labelled or those that expressed the corresponding fluorescent protein. The mouse monoclonal antibody NeuN reacts with a protein in cell nuclei from mouse brain^57^, subsequently identified as the splicing factor Fox-3^58^. The antibody apparently labels all neurons but no glial cells in rat spinal dorsal horn^59^, and the guinea pig NeuN antibody labels the same cells^42^. The NK1r antibody was raised in rabbit against a peptide corresponding to amino acids 393–407 of the rat NK1r, and has been validated by showing lack of staining in mice in which the gene is knocked out^60^. The affinity purified Homer antibody was raised against amino acids 1–175 of mouse Homer 1, and detects a band of the appropriate size in immunoblots of mouse brain extracts. We have shown that punctate staining with this antibody is associated with glutamatergic boutons in the spinal dorsal horn^43^. The pERK antibody is specific for ERK1 and ERK2 that are dually phosphorylated at Thr202 and Tyr204 sites. Specificity was shown by lack of staining in neurons in somatotopically appropriate areas after noxious stimulation.

### Statistical analysis

Statistical analyses were performed in Jamovi (The Jamovi project, Sydney, Australia) Categorical outcomes (e.g. NK1r status, retrograde labelling) were compared between groups by calculating proportions and retaining the animal as the biological unit. Differences in proportions were tested using ANOVA or paired t-tests as appropriate. The dependency of continuous outcomes (soma size, spine density) on categorical factors (Phox2a expression, pERK expression) were investigated using linear mixed models with individual cells clustered by animal^61^. Correlation was investigated using Pearson method.

## Supplementary Information

Supplementary Information.
