# Supplementary material for: Characterisation of lamina I anterolateral system neurons that express Cre in a Phox2a-Cre mouse line
Source: Sci Rep. 2021 Sep 9;11:17912. doi: 10.1038/s41598-021-97105-w (PMC8429737; doi:10.1038/s41598-021-97105-w)
Supplement: Supplementary file 1 — Supplementary Information. [file 41598_2021_97105_MOESM1_ESM.pdf]

Characterisation of lamina I anterolateral tract neurons that express Cre in a *Phox2a::Cre* mouse line

Wafa A. A. Alsulaiman[1]*, Raphaelle Quillet[1]*, Andrew M. Bell[1*], Allen C. Dickie[1], Erika Polgár[1], Kieran A. Boyle[1], Masahiko Watanabe[2], R. Brian Roome[3], Artur Kania[3], Andrew J. Todd[1] and Maria Gutierrez-Mecinas[1]

[1]Institute of Neuroscience and Psychology, College of Medical, Veterinary and Life Sciences, University of Glasgow, Glasgow, G12 8QQ, UK; [2]Department of Anatomy, Hokkaido University School of Medicine, Sapporo 060-8638, Japan; [3]Institut de Recherches Cliniques de Montréal (IRCM), Montréal, QC H2W 1R7, Canada

Number of words in	Abstract: 299
	Main text: 4947
	Online methods: 1887
Number of references: 62	
Number of figures:	11

Correspondence:

Andrew Todd, Spinal Cord Group, Sir James Black Building, University of Glasgow, Glasgow, G12 8QQ, UK. Email: andrew.todd@glasgow.ac.uk

or

Maria Gutierrez-Mecinas, Spinal Cord Group, Sir James Black Building, University of Glasgow, Glasgow, G12 8QQ, UK. Email: Maria.Gutierrez-Mecinas@glasgow.ac.uk

*These authors contributed equally

Fig S1

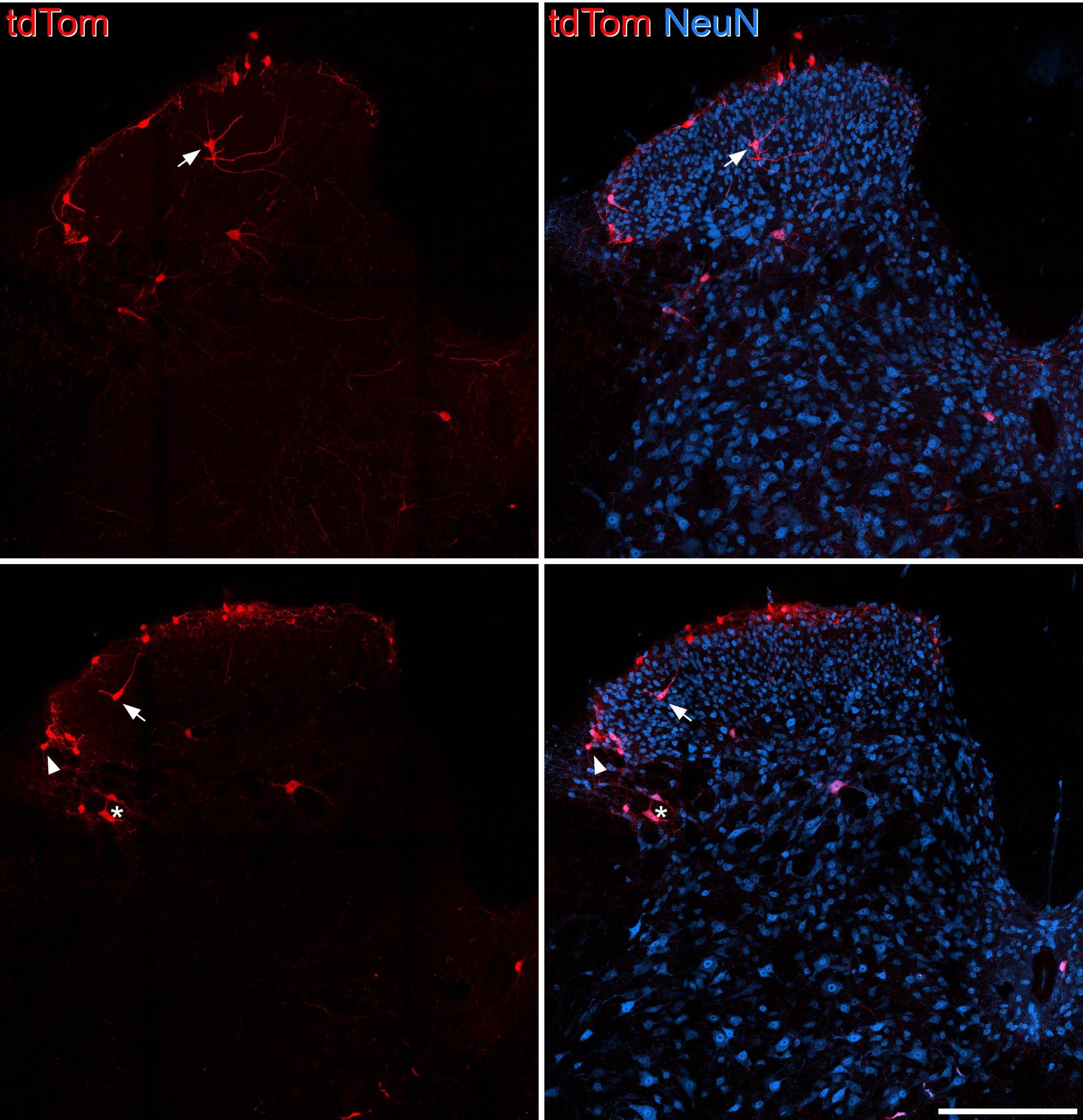

Fig S2

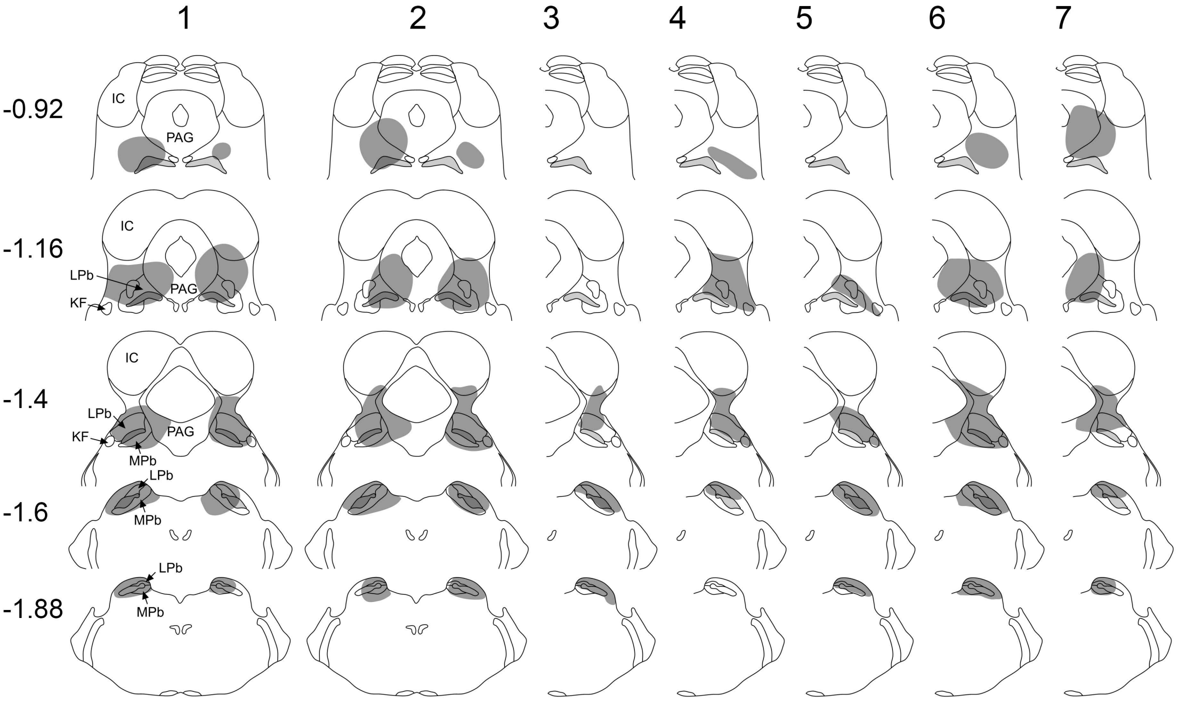

Fig S3

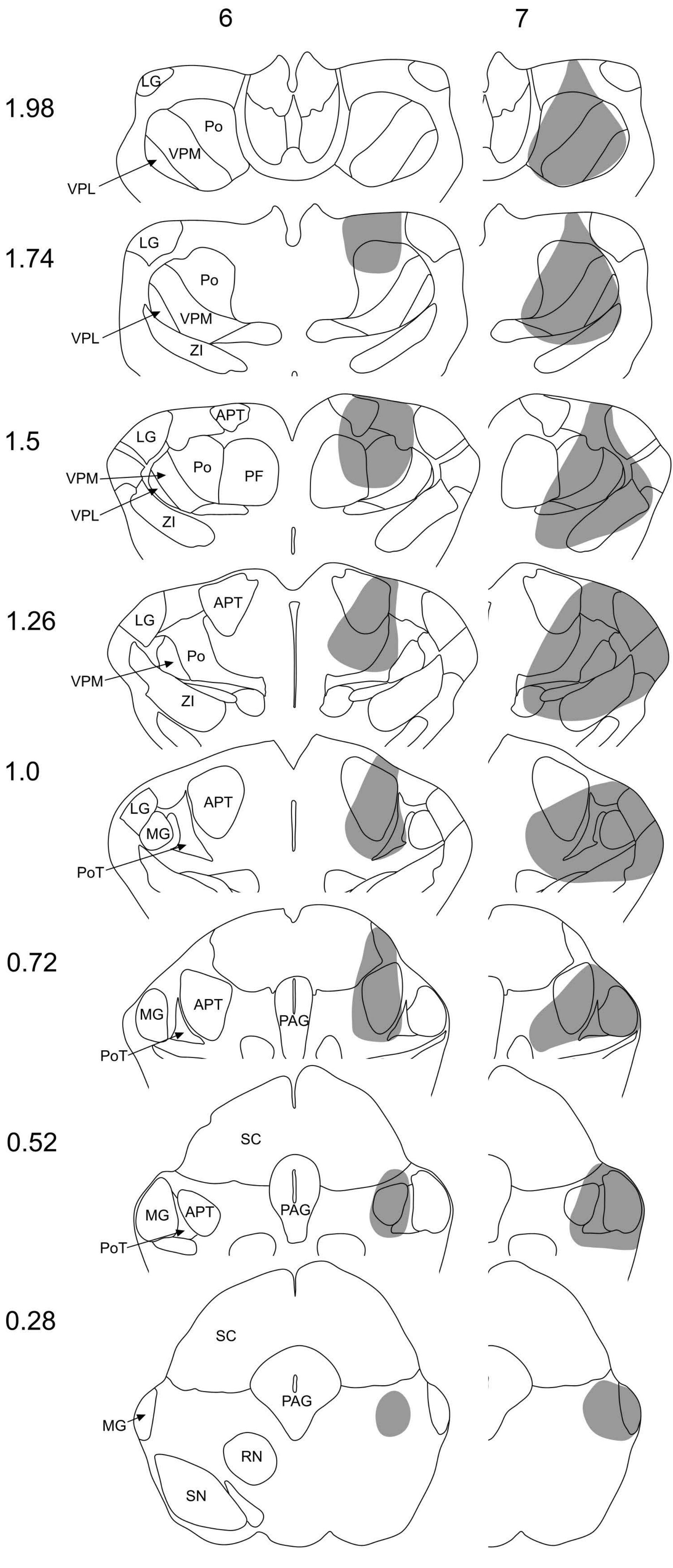
6
7
1.98
LG
Po
VPM
VPL
1.74
LG
Po
VPM
VPL
ZI
1.5
APT
LG
Po
PF
VPM
VPL
ZI
1.26
APT
LG
Po
VPM
ZI
1.0
APT
LG
MG
PoT
0.72
MG
APT
PAG
PoT
0.52
SC
MG
APT
PAG
PoT
0.28
SC
PAG
MG
RN
SN

Fig S4

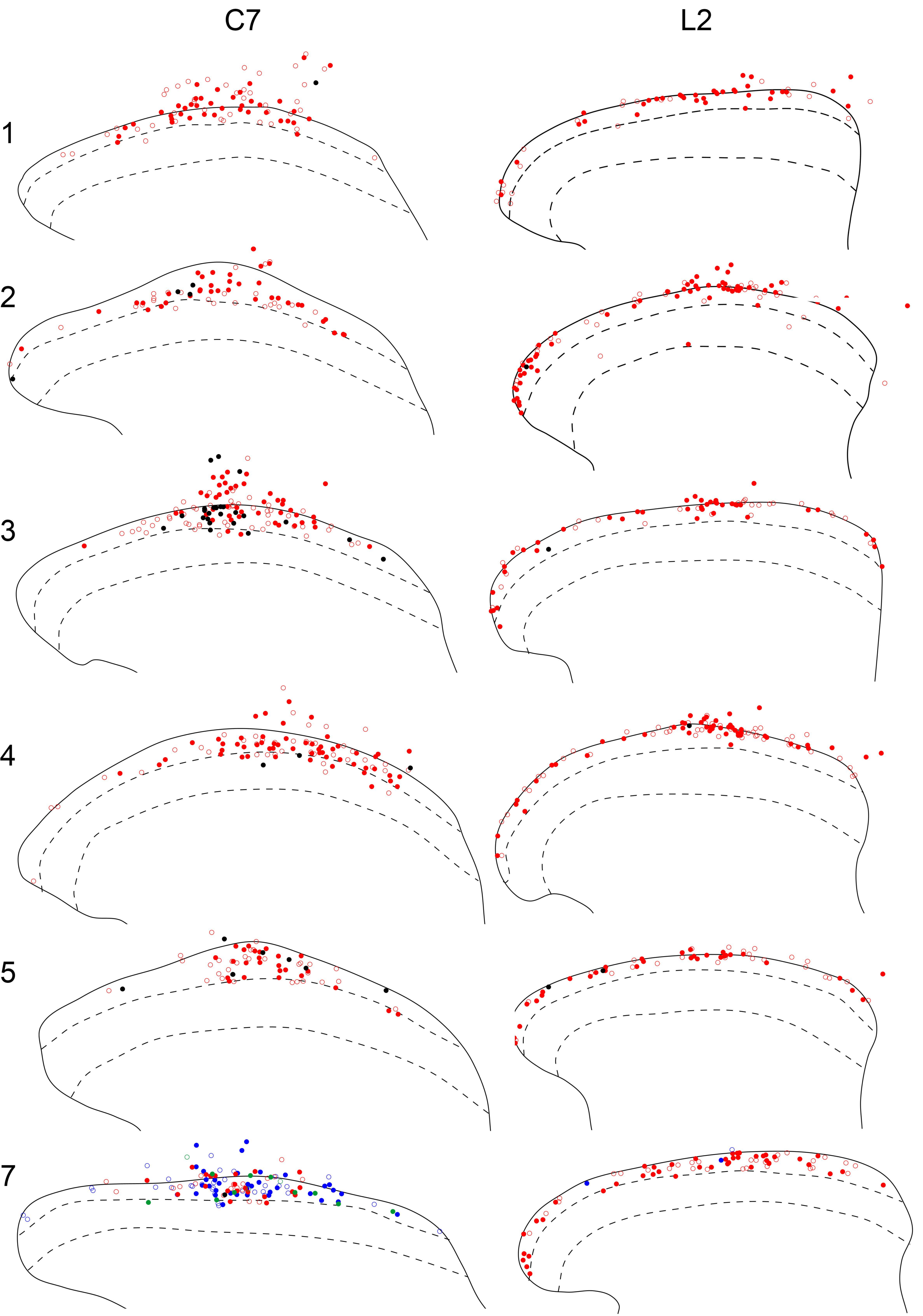

Table S1 Antibodies used in this study

Antibody	Species	Dilution	Source	Catalogue #
CTb	Goat	1:5K	List Biological	703
Fluorogold	Guinea Pig	1:500	Protos Biotech Corp	NM101
mCherry†	Chicken	1:5K	Abcam	ab205402
NeuN	Mouse	1:500	Millipore	MAB377
NeuN	Guinea Pig	1:500	Synaptic systems	266004
NK1r	Rabbit	1:20K*	Sigma-Aldrich	S8305
GFP	Chicken	1:1K	Abcam	ab13970
Homer	Goat	1:1K	Nittobo Medical	MSFR103230
pERK	Mouse	1:200	Cell Signaling Technology	9106

* Used with the tyramide signal-amplification method
†This antibody recognizes TdTomato

Table S2 RNAscope probes used in this study

**Probe**	**Protein/peptide**	**Channel numbers**	**Catalogue numbers**	**Z-pair number**	**Target region**
tdTomato	tdTom	1,2	317041	20	7 - 1382
Tac1	Substance P	3	410351	15	20 - 1034
Lypd1	Lypd1	1	318361	20	2318 - 1306
Tac1r	NK1r	2	428781	20	845 - 1775
Gpr83	Gpr83	3	317431	20	545 - 1578
RNAscope multiplex positive control (*Polr2a*, *Ppib*, *Ubc*)	Polr2a: DNA-directed RNA polymerase II subunit RPB1; Ppib: Peptidyl-prolyl cis-trans isomerase B; Ubc: Polyubiquitin-C	1,2,3	320881	20<br>15<br>n/a	2802 – 3678<br>98 – 856<br>34 - 860
RNAscope multiplex negative control (*dapB*)	dapB: 4-hydroxy-tetrahydrodipicolinate reductase (derived from B Subtilis)	1,2,3	320871	10	414 - 862

Supplementary figure legends

Fig S1 Distribution of tdTomato cells in the L2 segment in two *Phox2a::Cre;Rosa26*$^{LSL\text{-}tdTomato}$ mice. The sections have been stained to reveal tdTom (red) and NeuN (blue). In each case there are several tdTom-positive cells in lamina I, and scattered cells elsewhere in the grey matter. Cells in lamina III with long dorsal dendrites ("antenna cells") are indicated with arrows. There is a cluster of cells in the lateral, reticulated part of lamina V (asterisk), and occasional cells are seen in the lateral spinal nucleus (one indicated with an arrowhead). The images are projected from confocal scans (1 μm z-separation) through the full thickness of the section. Scale bar = 200 μm.

Fig S2 Diagrams to show the spread of CTb (dark shaded area) from the injection sites that were targeted on the lateral parabrachial area. Each vertical column represents a single experiment and the experiment number (corresponding to those in Tables 1-3) is shown at the top of the column. Numbers to the left show the approximate position of the section posterior to the ear-bar. Drawings are based on those in Franklin & Paxinos[62]. IC, inferior colliculus; KF, Kolliker-Fuse nucleus; LPb, lateral parabrachial area; MPb, medial parabrachial area; PAG, periaqueductal grey matter. The superior cerebellar peduncle is shown with light shading. Outline drawings were prepared with XaraXtreme v2 (http://www.xaraxtreme.org/).

Fig S3 Diagrams to show the spread of Fluorogold (shaded area) from the injection sites targeted on the thalamus. Each column represents a single experiment, with the number (corresponding to those in Tables 1-3) shown at the top. Numbers to the left show the approximate position of the section anterior to the ear-bar. Drawings are based on those in Franklin & Paxinos[62]. APT, anterior pretectal area; LG, lateral geniculate nucleus; MG, medial geniculate nucleus; PAG, periaqueductal grey matter;

PF, parafascicular nucleus; PoT, posterior triangular nucleus of the thalamus; RN, red nucleus; SC, superior colliculus; SN, substantia nigra; VPL, ventral posterolateral nucleus of thalamus; VPM, ventral posteromedial nucleus of thalamus; ZI, zona incerta. Outline drawings were prepared with XaraXtreme v2 (http://www.xaraxtreme.org/).

Fig S4 Plots showing the distribution of retrogradely-labelled and tdTomato-positive cells in the C7 and L2 segments from experiments #1-5 and #7. For experiments #1-5 cholera toxin B subunit (CTb) injections were targeted on the lateral parabrachial area. Red symbols show retrogradely labelled cells that were Phox2a-positive (filled) or Phox2a-negative (open). Phox2a cells that were not labelled with CTb are shown as solid black circles. In experiment #7 injections of CTb and Fluorogold were targeted on the lateral parabrachial area and thalamus, respectively. In this case tdTomato-positive and -negative cells are shown with solid and open symbols, respectively. Those that were retrogradely labelled with CTb and FG are shown in blue, and those labelled only with CTb in red and those labelled only with FG are green. tdTomato cells that were not retrogradely labelled are shown in black. Dashed lines represent the dorsal and ventral borders of lamina II. Outline drawings were prepared with XaraXtreme v2 (http://www.xaraxtreme.org/).
